# The Role of Membrane-Mediated Interactions in the Assembly and Architecture of Chemoreceptor Lattices

**DOI:** 10.1371/journal.pcbi.1003932

**Published:** 2014-12-11

**Authors:** Christoph A. Haselwandter, Ned S. Wingreen

**Affiliations:** 1 Departments of Physics & Astronomy and Biological Sciences, University of Southern California, Los Angeles, California, United States of America; 2 Department of Molecular Biology, Princeton University, Princeton, New Jersey, United States of America; Weizmann Institute of Science, Israel

## Abstract

*In vivo* fluorescence microscopy and electron cryo-tomography have revealed that chemoreceptors self-assemble into extended honeycomb lattices of chemoreceptor trimers with a well-defined relative orientation of trimers. The signaling response of the observed chemoreceptor lattices is remarkable for its extreme sensitivity, which relies crucially on cooperative interactions among chemoreceptor trimers. In common with other membrane proteins, chemoreceptor trimers are expected to deform the surrounding lipid bilayer, inducing membrane-mediated anisotropic interactions between neighboring trimers. Here we introduce a biophysical model of bilayer-chemoreceptor interactions, which allows us to quantify the role of membrane-mediated interactions in the assembly and architecture of chemoreceptor lattices. We find that, even in the absence of direct protein-protein interactions, membrane-mediated interactions can yield assembly of chemoreceptor lattices at very dilute trimer concentrations. The model correctly predicts the observed honeycomb architecture of chemoreceptor lattices as well as the observed relative orientation of chemoreceptor trimers, suggests a series of “gateway” states for chemoreceptor lattice assembly, and provides a simple mechanism for the localization of large chemoreceptor lattices to the cell poles. Our model of bilayer-chemoreceptor interactions also helps to explain the observed dependence of chemotactic signaling on lipid bilayer properties. Finally, we consider the possibility that membrane-mediated interactions might contribute to cooperativity among neighboring chemoreceptor trimers.

## Author Summary

The chemotaxis system allows bacteria to respond to minute changes in chemical concentration, and serves as a paradigm for biological signal processing and the self-assembly of large protein lattices in living cells. The sensitivity of the chemotaxis system relies crucially on cooperative interactions among chemoreceptor trimers, which are organized into intricate honeycomb lattices. Chemoreceptors are membrane proteins and, hence, are expected to deform the surrounding lipid bilayer, leading to membrane-mediated interactions between chemoreceptor trimers. Using a biophysical model of bilayer-chemoreceptor interactions we show that the membrane-mediated interactions induced by chemoreceptor trimers provide a mechanism for the observed self-assembly of chemoreceptor lattices. We find that the directionality of membrane-mediated interactions between trimers complements protein-protein interactions in the stabilization of the observed honeycomb architecture of chemoreceptor lattices. Our results suggest that the symmetry of membrane protein complexes such as chemoreceptor trimers is reflected in the anisotropy of membrane-mediated interactions, yielding a general mechanism for the self-assembly of ordered protein lattices in cell membranes.

## Introduction

The chemotaxis signal transduction pathway [1] allows bacteria to respond to minute relative changes in chemical concentration over several orders of magnitude in ambient chemical concentration [2], and ranks among the most studied signaling pathways in biology. The extreme sensitivity of the chemotaxis system results from amplification of external signals coupled with adaptation to persistent stimuli [3]–[7]. Fluorescence resonance energy transfer (FRET) experiments [8], [9] have revealed that a crucial step in signal amplification occurs at the level of chemoreceptors: chemoreceptors signal in cooperative teams [8]–[10]. Indeed, Ising [11]–[13] and Monod-Wyman-Changeux [9], [14]–[16] models of coupled teams of chemoreceptors both achieve quantitative agreement with the FRET data. The fundamental assumption underlying these models of signaling teams is that chemoreceptors do not respond independently to changes in the external ligand concentration, but rather each receptor influences the collective state of a team of neighboring receptors. Thus, the observed functional characteristics of chemotactic signaling rely on cooperative local interactions among chemoreceptors, and suggest a well-defined spatial organization of chemoreceptors.

From a structural perspective, chemoreceptors are homodimers, which interact strongly to form trimers-of-dimers [17], [18]. Recent breakthroughs in *in vivo* electron cryo-tomography have revealed [19]–[25] that chemoreceptor trimers form two-dimensional honeycomb lattices in which each trimer has three nearest-neighbors arranged in a face-on orientation. The honeycomb lattice architecture and characteristic lattice constant of 12 nm appear to be universally conserved among bacterial species [22]. Functional complexes require chemoreceptors plus the linker/kinase CheA and the linker protein CheW [19], [20], which may mediate cooperative interactions among neighboring trimers [9], [10]. Fluorescence experiments have indicated that chemoreceptor lattices can exhibit variable stoichiometries of chemoreceptors, CheA, and CheW [9], [26], [27], while clustering of chemoreceptors requires neither CheA nor CheW [27], [28]. The size of chemoreceptor clusters can range from tens to thousands of receptors, with large chemoreceptor clusters observed predominantly at the cell poles but smaller clusters also found in the midcell regions [21]–[25], [27]–[30]. Superresolution light microscopy of chemoreceptor lattices has suggested a stochastic model for cluster assembly [28], [30] in which self-assembly of chemoreceptor lattices proceeds by nucleation and growth. Such stochastic self-assembly of chemoreceptor lattices relies on the existence of attractive interactions between chemoreceptor trimers, but in principle does not require direct cytoskeletal involvement or active transport of chemoreceptors.

What are the molecular mechanisms yielding attraction between chemoreceptor trimers and, hence, self-assembly of chemoreceptor lattices? Chemoreceptors are transmembrane proteins localized in the cytoplasmic membrane of bacteria. In general, membrane proteins deform the surrounding lipid bilayer [31], [32], which can lead to membrane-mediated interactions between proteins [33], [34]. Thus, while chemoreceptors can be coupled by protein-protein interactions [9], [10], they may also interact via the cytoplasmic membrane. Here we develop a biophysical model of membrane-mediated interactions between chemoreceptor trimers which shows that membrane-mediated interactions can yield stochastic cluster assembly even at very dilute trimer concentrations. The model correctly predicts the observed face-on orientation of chemoreceptor trimers at small trimer separations [19], [20] and suggests a series of “gateway” states for chemoreceptor lattice assembly. We find that the three-fold-symmetric directionality of membrane-mediated interactions between trimers can stabilize the observed honeycomb architecture of chemoreceptor lattices [19], [20] even at suboptimal stoichiometries of chemoreceptors, CheA, and CheW [9], [26], [27]. The model also suggests a simple mechanism by which bilayer-chemoreceptor interactions can localize large chemoreceptor clusters to the cell poles even in the absence of interactions with CheA and CheW [27]. Furthermore, based on the assumption that the chemotactic signaling state impacts the hydrophobic thickness of chemoreceptors, our model allows us to quantify the membrane contribution to chemotactic signaling. In agreement with previous experimental observations [35]–[37] we find a dependence of chemotactic signaling on lipid bilayer properties. Finally, we examine the possibility of membrane-mediated cooperative signaling among neighboring chemoreceptor trimers.

## Models

**Figure 1:**
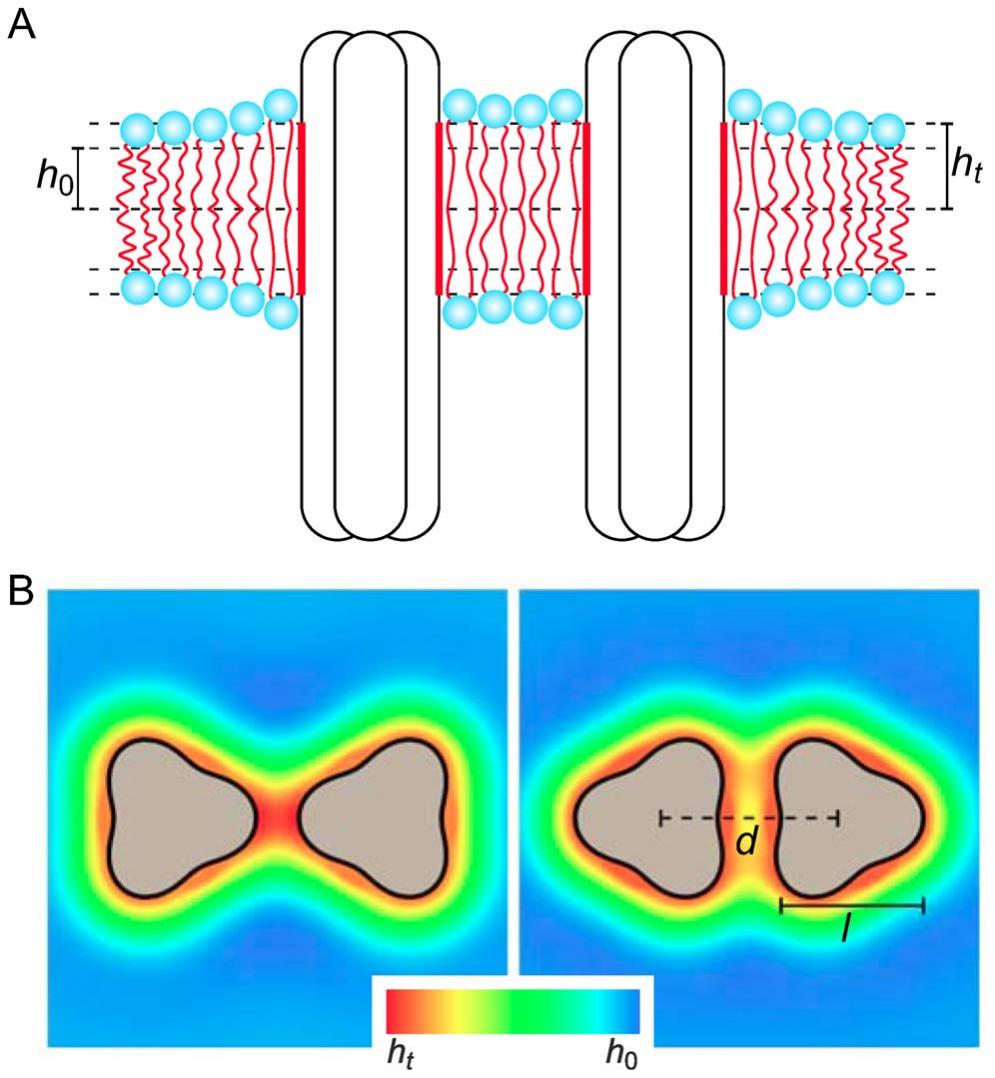
Schematic of membrane-mediated interactions between chemoreceptor trimers. (A) If the hydrophobic thickness of the unperturbed lipid monolayer, 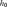, does not match the hydrophobic thickness of chemoreceptor trimers, 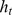, the lipid bilayer locally deforms in the vicinity of chemoreceptor trimers, yielding membrane-mediated interactions between trimers. (B) The three-fold symmetry of chemoreceptor trimers induces directionality of membrane-mediated interactions between trimers. The trimer configurations in the left and right panels correspond to tip-on and face-on orientations, respectively, with thickness deformations of the bilayer membrane in the vicinity of trimers illustrated by density maps (see S2 Figure for a three-dimensional illustration of thickness deformations). We denote by 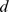 the center-to-center distance between trimers. In our calculations, we used chemoreceptor trimers of the indicated perturbed cylindrical shape with width 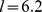 nm.

In our analysis of membrane-mediated interactions between chemoreceptor trimers we follow the standard membrane-mechanical framework [31]–[33] for describing bilayer-protein interactions, and model chemoreceptor trimers as rigid membrane inclusions inducing elastic deformations in the surrounding lipid bilayer membrane. Such deformations can take the form of thickness deformations (Fig. 1A), which originate from a hydrophobic thickness mismatch between chemoreceptors and the lipid bilayer, and midplane (curvature) deformations (S1 Figure), which may be induced by a conical shape of chemoreceptor trimers resulting from a tilt in the transmembrane helices. To leading order, the elastic energies associated with thickness and midplane deformations decouple from each other, and can therefore be analyzed separately (see S1 Text section 1). We focus here on bilayer-chemoreceptor interactions and, hence, only consider the transmembrane regions of trimers in our model, with the peri- and cytoplasmic regions of trimers in Fig. 1A and S1 Figure only being shown for illustration.

In general, neighboring membrane proteins are expected to induce overlapping deformation fields of the bilayer membrane, yielding [33], [34] membrane-mediated interactions between proteins. Thus, membrane proteins can interact over several nanometers [33], [34], [38] without being in direct protein-protein contact. Membrane-mediated interactions due to thickness/curvature deformations induced by identical proteins are generally expected [33] to be attractive/repulsive at small protein separations, with thickness deformations yielding stronger membrane-mediated interactions than curvature deformations. Based on previous work concerning the far-field limit of membrane-mediated interactions between conical membrane inclusions [39], [40], analytic series solutions describing membrane-mediated interactions between proteins of arbitrary symmetry and at arbitrary separation have recently been developed [41]. Here we employ these analytic series solutions to determine the membrane-mediated interactions between chemoreceptor trimers. We find that midplane interaction energies resulting from the conical shape of trimers are typically well below 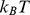 and more than an order of magnitude smaller than thickness interaction energies (see S1 Text section 2). We therefore focus on membrane-mediated interactions between chemoreceptor trimers due to thickness deformations (Fig. 1A).

Our biophysical model of membrane-mediated interactions between chemoreceptor trimers is based on the standard framework of membrane mechanics [42]–[46]. We represent the lipid bilayer within the Monge representation for curved surfaces using the functions 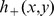 and 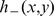, which define the heights of the hydrophilic-hydrophobic interfaces at the coordinates 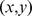 along the outer and inner membrane leaflets. The thickness deformations 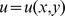 correspond to 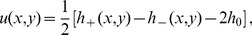
(1)where 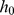 is one-half the hydrophobic thickness of the unperturbed lipid bilayer. Following previous work on bilayer-protein interactions [31]–[33], [47] we describe the energetic cost of thickness deformations by the functional 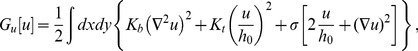
(2)where 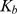 is the bending rigidity, 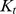 is the stiffness associated with thickness deformations, and 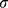 is the membrane tension. The term 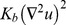 in Eq. (2) captures the energetic cost of membrane bending, while the term 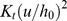 provides a simple description of the energetic cost of compressing or expanding the lipid bilayer. Typical measured values of 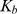 and 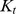 are 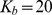 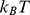 and 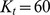 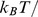
nm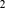 [33], [48], which we used for all the calculations described here. The term 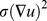 describes the effect of membrane tension on membrane undulations [42]–[47], [49], [50]. For generality we also allow for the term 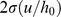 in Eq. (2) [38], [45], [48], which captures the effect of membrane tension on lipid surface area under conservation of lipid volume. Phenomenological membrane deformation energies of the form in Eq. (2) have been employed to describe protein-induced bilayer thickness deformations in a range of systems [31]–[33], [38], [46]–[55], and can be systematically refined [41], [56]–[68] to provide a more detailed model of bilayer-protein interactions.

The thickness deformation energy in Eq. (2) scales approximately with the square [31]–[33] of the hydrophobic mismatch which, in turn, is equal to the difference between one-half the trimer hydrophobic thickness, 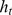, and 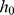. A typical value of 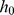 for the *E. coli* cytoplasmic membrane is 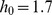 nm [69] while, for example, the approximate hydrophobic thickness of the chemoreceptor Trg is 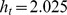 nm [70]. The resulting hydrophobic mismatch 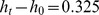 nm yields a thickness deformation energy of the order of 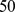 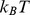 for a single chemoreceptor trimer, which induces strong membrane-mediated interactions between neighboring trimers (see the *Results* section). For a given value of 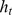, the value of 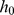 and hence the magnitude and sign of the hydrophobic mismatch, can be tuned by changing the membrane composition which, as demonstrated for gramicidin [47], [71] and mechanosensitive [49], [72] channels, allows for direct experimental tests of membrane-mechanical models of bilayer-protein interactions. We study here membrane-mediated interactions between chemoreceptor trimers as a function of hydrophobic mismatch. Thus, while we use in our calculations a chemoreceptor hydrophobic thickness consistent with Trg, our conclusions can be applied equally to other chemoreceptors.

In the absence of detailed structural information on the transmembrane region of chemoreceptor trimers, we adopt a highly simplified model designed to capture two key features of chemoreceptor trimers: (1) As described above, we assume that chemoreceptors have a hydrophobic mismatch with the lipid bilayer, which induces membrane-mediated interactions between neighboring chemoreceptor trimers (Fig. 1A). (2) In addition, the characteristic three-fold symmetry of chemoreceptor trimers yields directionality in membrane-mediated interactions between chemoreceptor trimers (Fig. 1B). In particular, different relative orientations of neighboring chemoreceptor trimers produce distinct deformations of the bilayer membrane, resulting in a dependence of the energy of membrane-mediated interactions on the relative trimer orientation. Thus, membrane-mediated interactions between chemoreceptor trimers not only depend on the hydrophobic mismatch between chemoreceptors and the bilayer membrane [33], but also on the distinctive three-fold symmetry of chemoreceptor trimers. While the precise size of the transmembrane cross section of trimers is not crucial for our model predictions, we allow for a finite characteristic size of trimers and lipids, which imposes steric constraints on the minimum edge-to-edge separation of neighboring trimers.

Our simple model for the shape of chemoreceptor trimers (Fig. 1B) is consistent with recent electron cryo-tomography studies [19], [20]. However, we focus here on the effects of generic aspects of chemoreceptor trimers, such as their symmetry, on membrane-mediated interactions, and our predictions do not rely on the detailed supramolecular shape of trimers. In particular, data obtained from electron cryo-tomography [19], [20], [73] mostly pertains to the cytoplasmic regions of chemoreceptor trimers, and the transmembrane structure of chemoreceptor trimers remains unknown. Indeed, the chemoreceptor dimers forming a trimer may spread apart within the membrane [19], [20], [73], with the lipid bilayer infiltrating chemoreceptor trimers. Such lipid-chemoreceptor complexes would imply membrane-mediated interactions between the chemoreceptor dimers forming a trimer. Here we do not consider membrane-mediated interactions within trimers and, instead, focus on membrane-mediated interactions between chemoreceptor trimers. Thus, our model of the transmembrane shape of chemoreceptor trimers (Fig. 1B) may correspond to chemoreceptor trimers composed of only proteins as well as lipid-chemoreceptor complexes. For simplicity, we assume a constant hydrophobic thickness of chemoreceptor trimers. More detailed descriptions would allow for a variation of the hydrophobic thickness along the trimer circumference, which may result from details of the transmembrane structure of chemoreceptors or the formation of lipid-chemoreceptor complexes.

Multiple lines of evidence [74]–[78] have indicated that chemoreceptor dimers signal the binding of a ligand across the cytoplasmic membrane through a piston-like sliding of one of the four transmembrane helices relative to the other three helices, by approximately 0.16 nm. This suggests that chemotactic signaling perturbs the hydrophobic surface of chemoreceptors and, indeed, it has been found [35]–[37] that bilayer-chemoreceptor interactions affect chemotactic signaling. Furthermore, the *in vivo* signaling response of chemoreceptors implies [9], [16] that trimers exhibit strong cooperativity, and are either in the fully active or the fully inactive state. We account for these observations by assuming that chemoreceptor trimers can be active or inactive, with active and inactive trimers exhibiting a difference in hydrophobic thickness. For simplicity, we also assume that this difference in hydrophobic thickness is uniform along the trimer circumference, and is approximately equal to 0.16 nm as indicated by the piston model of chemotactic signaling [74]–[78]. (While consistent with the observed role of the membrane in chemotactic signaling, this working model is highly simplified; more detailed models would allow, for instance, for the possibility of a tilt in the transmembrane helices upon switching [74], [79], for variations in the shift in hydrophobic thickness along the trimer circumference, and for possible differences in the hydrophobic surfaces exhibited by distinct chemoreceptors.) The predicted strength of the coupling between bilayer properties and chemotactic signaling depends on model details, but the basic mechanism for membrane-mediated cooperativity among chemoreceptors considered here relies only on a difference in hydrophobic thickness between active and inactive trimer states.

## Results

### Nucleation and growth of chemoreceptor lattices

**Figure 2:**
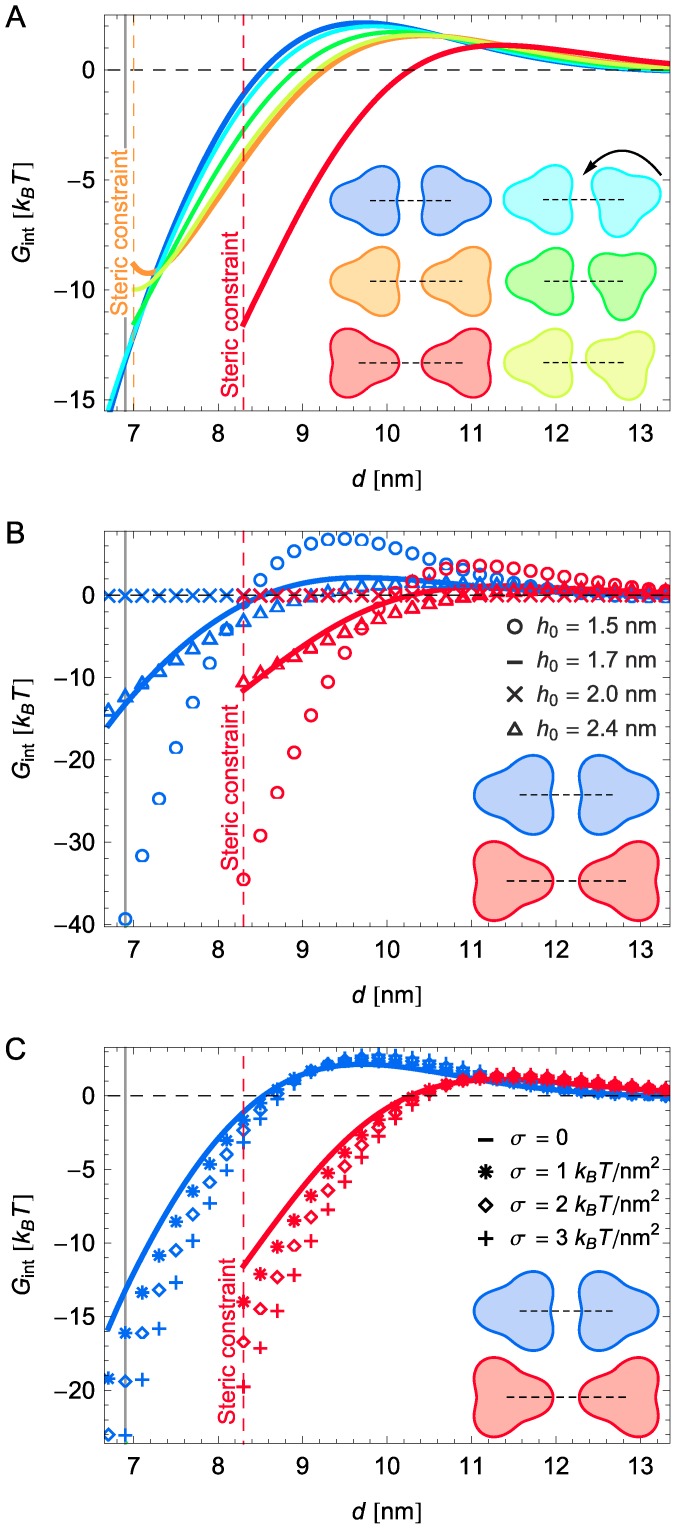
Membrane-mediated interactions yield attraction between chemoreceptor trimers. Calculated elastic interaction potentials between chemoreceptor trimers, 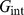, as a function of center-to-center distance between neighboring trimers and (A) trimer orientation, (B) lipid monolayer hydrophobic thickness, and (C) membrane tension, 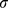. The solid lines at 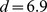 nm indicate the face-on trimer separation measured by electron cryo-tomography of chemoreceptor lattices in the presence of CheA and CheW [19], [22] and, where appropriate, steric constraints on trimer configurations due to finite trimer and lipid size are indicated by dashed vertical lines marking the end points of interaction potentials. For (A) and (B) we set 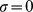, and for (A) and (C) we used the monolayer thickness 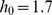 nm corresponding to the *E. coli* cytoplasmic membrane. All interaction potentials were calculated analytically under the approximation that trimers are weakly perturbed cylindrical inclusions.

We followed the approach developed in Refs. [39]–[41] to obtain analytic expressions for the energy 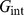 of membrane-mediated interactions between chemoreceptor trimers due to thickness deformations. The energy 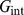 is a function of center-to-center distance between trimers, 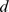, relative trimer orientation, membrane tension, and hydrophobic mismatch (see S1 Text section 1). A negative value of the energy of membrane-mediated interactions, 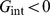, implies energetically favorable interactions between chemoreceptor trimers. For a hydrophobic mismatch corresponding to chemoreceptors and the cytoplasmic membrane of *E. coli*, we find three regimes of membrane-mediated interactions between chemoreceptor trimers (Fig. 2A): (1) For trimer separations greater than 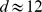 nm membrane-mediated interactions are negligible, yielding energies smaller than 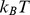. (2) For intermediate trimer separations, from 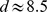
–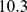 nm (depending on relative trimer orientation) up to 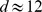 nm, interactions are weakly unfavorable. (3) For small trimer separations, 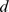 smaller than 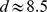
–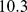 nm (depending on relative trimer orientation), membrane-mediated interactions are strongly favorable. In particular, we find that for the smallest values of 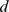 allowed by steric constraints on lipid size, corresponding to a minimum edge-to-edge separation between trimers of approximately 0.8 nm, membrane-mediated interactions can reduce the thickness deformation energy by more than 15 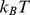 compared to noninteracting chemoreceptor trimers.

The interaction potentials in Fig. 2A show that membrane-mediated interactions yield strong attraction between chemoreceptor trimers over several nanometers, which suggests that membrane-mediated interactions may be sufficient for nucleation and growth of chemoreceptor lattices. Indeed, while chemoreceptors interact with CheA and CheW to form ordered lattices [19], [20], clustering of chemoreceptors requires neither CheA nor CheW [27], [28]. Furthermore, superresolution light microscopy of chemoreceptor clusters has suggested [28], [30] that chemoreceptor lattices self-assemble by stochastic nucleation of small clusters and capture of diffusing receptors by preexisting clusters. Fig. 2A implies that membrane-mediated interactions provide a plausible biophysical mechanism for the efficient self-assembly of chemoreceptor lattices via stochastic nucleation and capture. In particular, based on the statistical mechanics of phase segregation [80], [81] the interaction energies in Fig. 2A allow us to estimate the critical trimer concentration for nucleation and growth of chemoreceptor lattices in the *E. coli* cytoplasmic membrane (see S1 Text section 3). We find that the critical trimer concentration for clustering is already reached with approximately 15 chemoreceptor trimers in the cytoplasmic membrane. This means that, even if trimers are very dilute in the cytoplasmic membrane, membrane-mediated interactions can lead to nucleation and growth of chemoreceptor lattices.

Our model predicts that chemoreceptor clustering due to membrane-mediated interactions shows a characteristic dependence on hydrophobic mismatch (Fig. 2B) and membrane tension (Fig. 2C). In Fig. 2B we consider a range in hydrophobic mismatch which may be realized, for instance, by varying the tail lengths in phosphatidylcholine (PC) lipid bilayers from PC10 to PC24 [82], while in Fig. 2C we consider values of membrane tension up to the approximate rupture tension of lipid bilayers [48], [82]. Fig. 2B shows that membrane-thickness-mediated interactions between chemoreceptor trimers vanish when the bilayer hydrophobic thickness matches the chemoreceptor hydrophobic thickness, and increase in magnitude with increasing magnitude of hydrophobic mismatch. Fig. 2C predicts that, for a hydrophobic mismatch corresponding to chemoreceptors and the cytoplasmic membrane of *E. coli*, membrane-mediated interactions between chemoreceptor trimers become more pronounced with increasing membrane tension, yielding an increased propensity for chemoreceptor clustering.

The basic qualitative features of the interaction potentials in Fig. 2 can be understood from the thickness deformation field due to a single membrane inclusion. Consider, for simplicity, a cylindrical membrane inclusion with a hydrophobic thickness that exceeds the unperturbed bilayer hydrophobic thickness. The resulting thickness deformation decays approximately exponentially around the membrane inclusion with a characteristic decay length 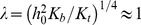 nm [33], [48]. The decaying thickness deformation will overshoot [47], [52], leading to a zone of compression of the lipid bilayer, before the deformation eventually approaches zero (S3 Figure). The attractive regime of membrane-mediated interactions in Fig. 2 corresponds to edge-to-edge separations of up to approximately 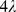, for which thickness deformations mainly overlap in the region of initial exponential decay and the overall deformation footprint of the two trimers is reduced compared to noninteracting trimers (Fig. 1A). For edge-to-edge separations from approximately 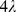 to 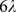, the compressed and expanded membrane regions induced by the two trimers strongly overlap, which results in frustration of membrane deformations and the repulsive regime in Fig. 2. Finally, the noninteracting regime in Fig. 2 corresponds to edge-to-edge separations greater than approximately 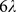, for which there is only little overlap in the thickness deformations induced by the two trimers. The scale of the maximum interaction energies in Fig. 2 is set by the single-cylinder thickness deformation energy 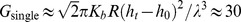 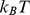 [33] for a radius *R* = 3.1 nm. Also, since 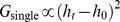, the strength of the attractive and repulsive regimes increases with the magnitude of the hydrophobic mismatch as in Fig. 2B. Moreover, the single-inclusion thickness deformation energy increases with membrane tension if, as is the case for chemoreceptors in the cytoplasmic membrane, the hydrophobic mismatch takes a positive value [48], yielding an increase in the strength of membrane-mediated interactions with increasing membrane tension as in Fig. 2C.

### Gateway to chemoreceptor lattice architecture

Fig. 2 shows that membrane-mediated interactions between chemoreceptor trimers are strongly directional, and reflect the three-fold symmetry of trimers. We find two dominant trimer configurations as a function of trimer separation: (1) In Fig. 2A, for trimer separations greater than 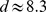 nm, the tip-on configuration (red inset) is energetically most favorable. (2) For small trimer separations, 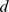 smaller than 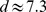 nm, the face-on configuration (blue inset) is most favorable. These two regimes occur because the tip-on configuration yields the smallest edge-to-edge separation (and thus the longest-range interactions, Fig. 1B left panel), while the face-on configuration maximizes the membrane area over which trimer-induced thickness deformations can overlap (and thus provides the maximum interaction strength overall, Fig. 1B right panel). We estimate that the energy difference between tip-on and face-on configurations can be more than 10 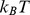 for the minimum trimer separations allowed by steric constraints in the two configurations. In particular, membrane-mediated interactions favor the face-on trimer configuration at the observed separation 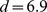 nm (grey vertical line) as measured by electron cryo-tomography of chemoreceptor lattices in *E. coli* as well as other organisms [19], [22], in the presence of CheA and CheW. The face-on configuration of trimers predicted by our model for small trimer separations has been observed in chemoreceptor lattices in a variety of different organisms [19], [20] and allows the formation of chemoreceptor-CheW-CheA complexes, yielding a well-defined trimer separation due to direct protein-protein interactions.

**Figure 3:**
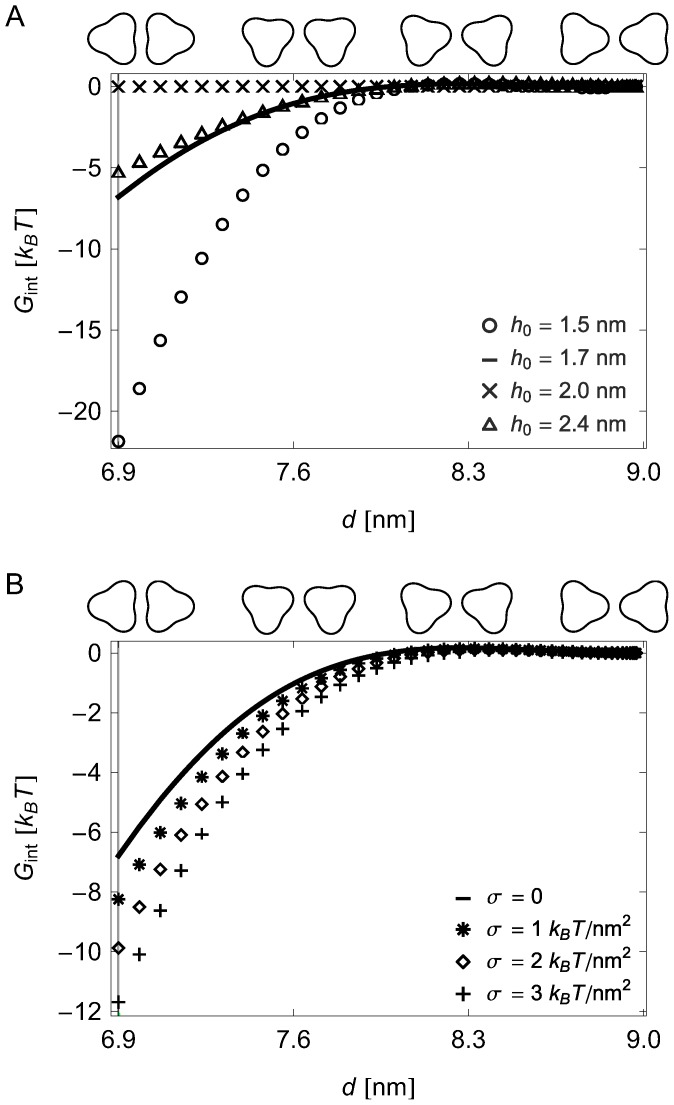
Gateway to assembly of face-on trimer configuration. Calculated elastic interaction energy between two chemoreceptor trimers as a function of trimer orientation (upper axes) and center-to-center trimer distance (lower axes), and (A) membrane thickness and (B) membrane tension. Trimer configurations are rotated from the tip-on to the face-on configuration while maintaining reflection symmetry and a minimum edge-to-edge separation of 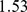 nm, which yields the face-on trimer configuration at 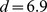 nm (and the tip-on trimer configuration at 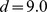 nm). The vertical lines at 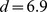 nm indicate the face-on trimer separation measured for chemoreceptor lattices [19], [22]. For ease of comparison, the zero energy for each curve was set at the tip-on configuration. For (A) we set 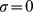 and for (B) we used the monolayer thickness 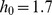 nm corresponding to the *E. coli* cytoplasmic membrane. All trimer interaction energies were calculated as in Fig. 2.

Fig. 2 implies a scenario for the assembly of chemoreceptor lattices in which the tip-on trimer configuration is a gateway state yielding attraction between chemoreceptor trimers over several nanometers, with the directionality of membrane-mediated interactions ensuring that, at small separations, trimers are arranged in the face-on orientation allowing further stabilization through direct protein interactions mediated by CheA and CheW [19], [20]. In particular, the interaction potentials in Fig. 2 suggest that the face-on trimer configuration found in chemoreceptor lattices [19], [20] could be achieved through the sequence of gateway states shown in Fig. 3. For large 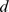, the tip-on configuration is strongly favored (for ease of visualization, the tip-on configuration is set as the zero of 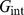 in Fig. 3). As the trimer separation shrinks below the steric constraint on the tip-on configuration, the membrane deformation energy can be lowered further by a symmetric rotation of the chemoreceptor trimers (S1 Video), ultimately yielding the observed face-on trimer configuration [19], [20] as the lowest-energy configuration, thus ensuring correct assembly of chemoreceptor lattices. Consistent with the results in Fig. 2, we find that the membrane-mediated interactions stabilizing the sequence of gateway states in Fig. 3 vanish for lipid bilayers matching the chemoreceptor hydrophobic thickness and increase with the magnitude of the hydrophobic mismatch (Fig. 3A). Similarly, our model predicts that the reduction in membrane deformation energy associated with the sequence of gateway states in Fig. 3 increases with increasing membrane tension (Fig. 3B).

### Stabilization of chemoreceptor lattice architecture

**Figure 4:**
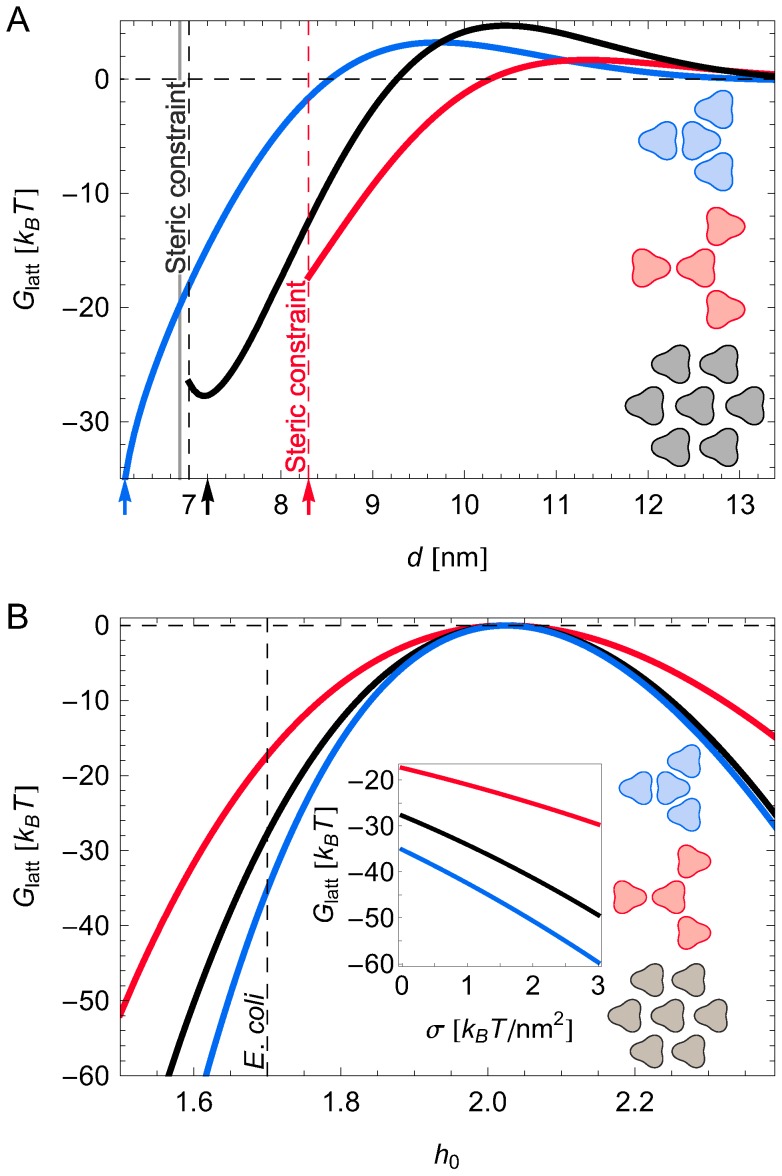
Membrane-mediated interactions yield the observed architecture of chemoreceptor lattices. Calculated elastic interaction energy per trimer, 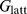, in face-on honeycomb (blue), tip-on honeycomb (red), and hexagonal (black) lattices as a function of (A) center-to-center distance between neighboring trimers and (B) monolayer hydrophobic thickness and membrane tension (inset) at 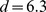 nm (face-on honeycomb lattice; blue curve), 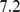 nm (hexagonal lattice; black curve), and 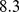 nm (tip-on honeycomb lattice; red curve), corresponding to the lattice spacings indicated by arrows in (A). The solid vertical line at 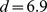 nm in (A) shows the trimer separation observed in face-on chemoreceptor lattices [19], [22], and dashed vertical lines in (A) indicate steric constraints on tip-on honeycomb (red) and hexagonal (black) lattice configurations. For (A) we assumed a membrane with 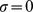 and a monolayer thickness 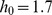 nm corresponding to the *E. coli* cytoplasmic membrane (shown by a dashed vertical line in (B)). For the main panel in (B) we set 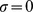 and the curves in the inset were obtained with 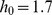 nm. All lattice energies were calculated from pairwise nearest-neighbor interaction potentials as shown in Fig. 2.

A simple arrangement of trimers in chemoreceptor lattices would be a close-packed hexagonal lattice structure (Fig. 4 grey insets, S4A Figure) in which each trimer has six nearest neighbors and, hence, the number of nearest-neighbor interactions is maximized. However, electron cryo-tomography has shown [19], [20] that chemoreceptor trimers are not closely packed in chemoreceptor lattices but rather form a honeycomb lattice in which each trimer has three nearest-neighbors arranged in the face-on orientation (Fig. 4 blue insets, S4B Figure), which allows formation of an extended lattice composed of chemoreceptor trimers, CheA, and CheW. To elucidate the stability of the observed face-on honeycomb-lattice architecture we calculated the energy per chemoreceptor trimer resulting from membrane-mediated interactions due to thickness deformations, 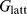, in face-on honeycomb, tip-on honeycomb (Fig. 4 red insets, S4C Figure), and hexagonal lattices. We find that, while tip-on honeycomb and hexagonal lattices can be energetically favorable for large lattice spacings, both these structures are unstable to the formation of a face-on honeycomb lattice with small lattice spacing, which provides the minimum-energy lattice architecture (Fig. 4A). This conclusion is robust with respect to variations in hydrophobic mismatch (Fig. 4B) and membrane tension (Fig. 4B inset). In contrast, cylindrical membrane inclusions, which do not exhibit directional interactions, would yield the hexagonal lattice as the minimum-energy structure. Thus, the directionality of membrane-mediated interactions stabilizes the observed face-on honeycomb lattice architecture against the tip-on honeycomb and hexagonal lattice structures. Specifically, the three-fold symmetry of trimers allows honeycomb ordering of chemoreceptor lattices, and thus further stabilization of a well-defined lattice constant through direct protein interactions with CheA and CheW [19], [20].

Fig. 4B predicts that, for the lattice spacings indicated by arrows in Fig. 4A, the strength of favorable interactions between chemoreceptor trimers in face-on honeycomb, tip-on honeycomb, and hexagonal lattices grows monotonically with increasing hydrophobic mismatch between lipid bilayer and chemoreceptors, as well as with increasing membrane tension. For the lattice spacings in Fig. 4A yielding a crossover from favorable (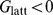) to unfavorable (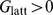) lattice energies we obtain a more complex dependence of the lattice energy on bilayer hydrophobic thickness and membrane tension (S5 Figure). In particular, for such crossover lattice spacings our model predicts favorable lattice energies for bilayer hydrophobic thicknesses exceeding the chemoreceptor hydrophobic thickness, with unfavorable lattice energies for bilayer hydrophobic thicknesses smaller than the chemoreceptor hydrophobic thickness. This can be understood by noting that the decay length 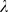 increases with 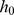, thus shifting membrane-mediated interactions into the attractive regime if 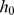 increases beyond 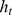, and *vice versa*. Finally, we note that the lattice energy due to membrane-mediated interactions between chemoreceptor trimers is dominated by nearest-neighbor interactions, with longer-range interactions only yielding minor shifts in the lattice energy (S6 Figure).

### Transition in chemoreceptor lattice architecture

Our calculations imply that close-packed hexagonal lattices of chemoreceptor trimers are metastable in the sense that the hexagonal lattice structure is only a local minimum of the membrane deformation energy, with the global minimum provided by the face-on honeycomb lattice (Fig. 4). However, the membrane area per trimer in honeycomb lattices is greater than the membrane area per trimer in hexagonal lattices—by 50% if both lattice structures have the same trimer separation and by 15% for the trimer separations indicated by arrows in Fig. 4. Thus, in situations where the clustering of chemoreceptor trimers is strongly constrained by the available membrane area, membrane-mediated interactions may yield hexagonal chemoreceptor lattices. On the basis of electron microscopy it has indeed been observed [83]–[85] that overexpression of chemoreceptors results in hexagonal lattices of trimers in the cytoplasmic membrane. The observed two-dimensional hexagonal lattices were distinct from the “zippered” cluster structures [83] also found in overexpression experiments, which strongly bend the membrane and form interdigitated protein contacts. In agreement with our model, in the case of overexpression the clustering of trimers, the stability of the lattice, and the two-dimensional hexagonal lattice architecture did not rely on the presence of CheA and CheW, although the presence of CheA and CheW yielded more ordered lattice structures and modified the lattice spacing [84], [85]. The trimer orientation in the observed two-dimensional hexagonal lattices [84], [85] is consistent with the hexagonal lattice architecture of trimers shown in Fig. 4 (grey insets).

### Localization of chemoreceptor lattices to cell poles

As noted in the *Models* section, chemoreceptor trimers induce midplane deformations in addition to thickness deformations. While midplane interaction energies are typically negligible compared to thickness interaction energies (see S1 Text section 2), midplane deformations provide a simple mechanism for segregation of chemoreceptor trimers to the cell poles [86]. In particular, the energetic cost of trimer-induced curvature deformations depends on the interplay between the conical shape of chemoreceptor trimers [87] and the preferred curvature of the surrounding lipid bilayer: Since the average membrane radius of curvature at the poles of *E. coli* is approximately twice that of the midcell region, and both have the same sign as the radius of curvature of chemoreceptor trimers, midplane deformations may act as curvature sensors mediating localization of chemoreceptor trimers to the cell poles.

The energy of trimer-induced midplane deformations can be estimated using a variety of different approaches [48], [50], [80], [81]. Independent of the particular model formulation, we find that for a single chemoreceptor trimer the difference in midplane deformation energy between the poles and midcell of *E. coli* is well below 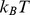 (see S1 Text section 2). This suggests that curvature deformations are not able to localize individual chemoreceptor trimers to the cell poles. However, as described above, we also find that strong membrane-mediated interactions due to thickness deformations effectively bind chemoreceptor trimers into chemoreceptor lattices, which may be further stabilized by interactions with CheA and CheW. For a lattice composed of 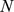 chemoreceptor trimers we estimate an energy difference 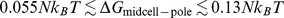
(3)between the midcell and poles of *E. coli* in the regime of weak interactions due to midplane deformations, where the lower and upper bounds correspond to different model formulations (see S1 Text section 2). Thus, bilayer-trimer interactions yield only weak curvature sensitivity for small chemoreceptor lattices but can readily induce localization of large chemoreceptor lattices to convex regions of the cytoplasmic membrane such as the cell poles. Large chemoreceptor lattices composed of thousands of receptors (for which Eq. (3) yields 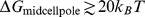) are indeed observed predominantly at the cell poles, while smaller chemoreceptor lattices are also found in the midcell regions [21]–[25], [27]–[30].

### Effects of bilayer-chemoreceptor interactions on chemotactic signaling

Reconstitution of chemoreceptors in bilayer vesicles [36] and nanodiscs [35] has indicated that the signaling properties of chemoreceptors depend on the composition of lipid bilayers. Furthermore, modification of the transmembrane properties of chemoreceptors by site-directed mutagenesis has shown [37] that bilayer-chemoreceptor interactions influence chemotactic signaling. Within the simple “piston” model of chemotactic signaling such a coupling between chemoreceptor function and lipid bilayer properties arises naturally—specifically, the on and off states of chemoreceptors differ in their hydrophobic mismatch with the lipid bilayer and thus in their bilayer deformation energies. Assuming a uniform 0.16 nm difference in hydrophobic thickness between on and off states, our model predicts that for *E. coli* the membrane contribution to the total free-energy difference between on and off states of a single chemoreceptor trimer is greater than 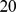 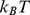 in magnitude, and can vary over more than 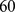 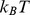 with bilayer or trimer hydrophobic thickness (S7 Figure). In agreement with experiments [35]–[37] we therefore find that shifts in the membrane contribution to the trimer transition energy due to modification of lipid composition or chemoreceptor transmembrane properties can dominate over shifts in the transition energy due to chemoreceptor methylation, which are of the order of 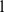 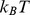 per methyl group [88]. In addition, our model predicts a dependence of chemotactic signaling on membrane elastic properties such as membrane tension (S7 Figure inset). In particular, we find that variation in membrane tension can shift the membrane contribution to the on-off transition energy by up to 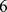 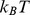 which, again, is comparable to shifts in the trimer transition energy due to chemoreceptor methylation [88].

**Figure 5:**
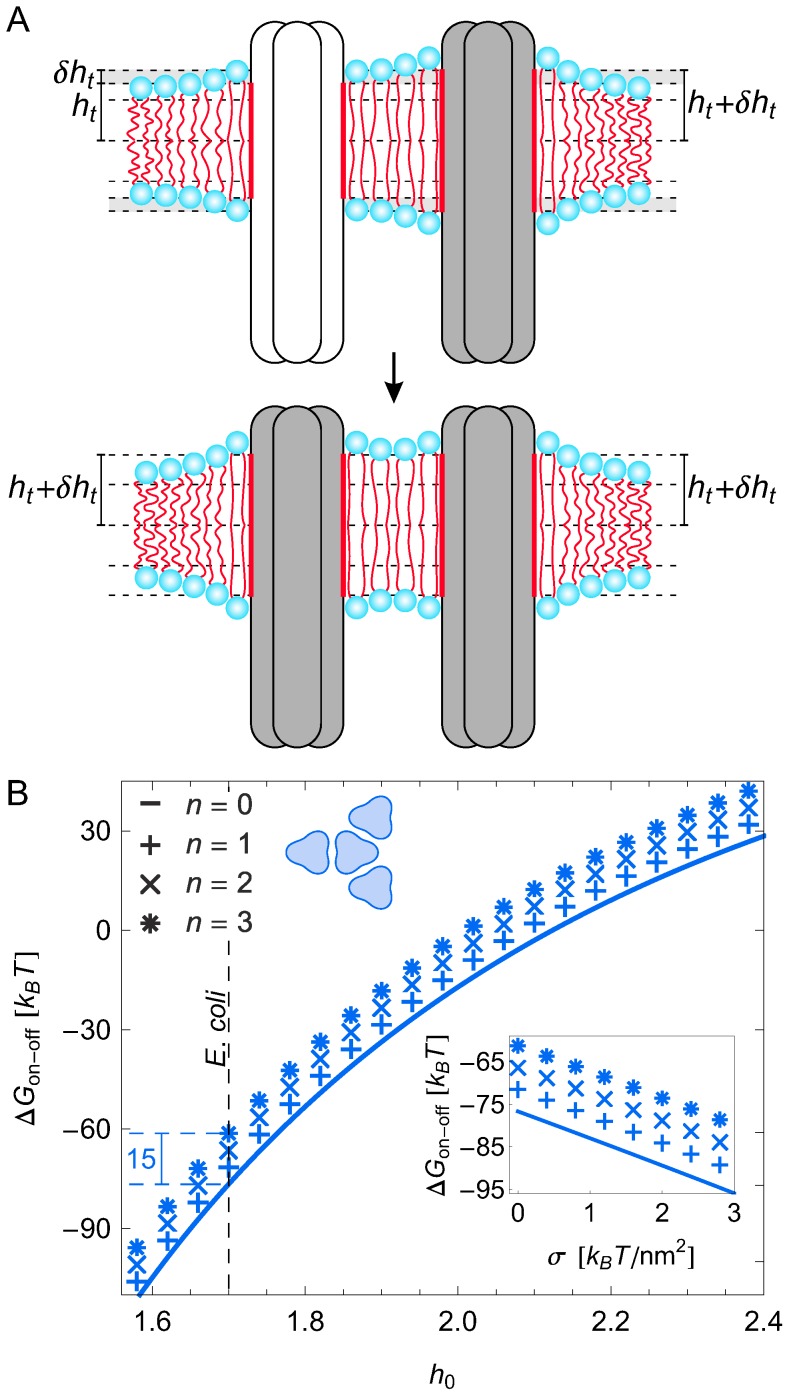
Membrane-mediated interactions may contribute to cooperative signaling. (A) We assume the trimer hydrophobic thickness differs by 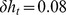 nm between the on state (white trimers, 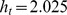 nm) and the off state (grey trimers, 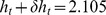 nm) according to the piston model of chemotactic signaling. For ease of visualization, the magnitude of 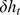 is exaggerated in the schematic illustrations of the off state. (B) Calculated difference in the thickness deformation energy associated with the on and off states of a chemoreceptor trimer, 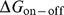, in a face-on honeycomb lattice with 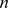 nearest neighbors in the off state as a function of membrane hydrophobic thickness and membrane tension (inset) at the trimer spacing 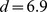 nm measured for face-on chemoreceptor lattices [19], [22]. For the main panel the membrane tension is 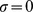 and for the inset the monolayer hydrophobic thickness is 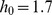 nm corresponding to the *E. coli* cytoplasmic membrane (indicated by a dashed vertical line in the main panel, with the dashed horizontal lines showing 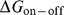 for 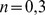 neighboring trimers in the off state). All cooperative interactions were calculated using pairwise trimer-trimer interaction potentials as in Fig. 4.

We speculate that membrane-mediated interactions between chemoreceptor trimers could contribute to cooperativity among chemoreceptor trimers, complementing the contribution of direct protein interactions mediated by CheA and CheW [9], [10], [19], [20]: Consider a chemoreceptor trimer in the on state, with a neighboring trimer in the off state (Fig. 5A upper panel). Assuming that the two trimers induce distinct thickness deformations due to their different signaling states, membrane-mediated interactions are energetically unfavorable at small trimer separations [33]. If, however, both trimers are in the off state (Fig. 5A lower panel), membrane-mediated interactions are strongly favorable. Thus, the presence of a neighboring trimer in the off state lowers, via membrane-mediated interactions, the free energy of the off state (and similarly a neighbor in the on state lowers the free energy of the on state), potentially yielding membrane-mediated cooperativity among chemoreceptor trimers.

In order to quantify the above mechanism for membrane-mediated cooperativity we calculated the membrane contribution to the free-energy difference between the on and off state of a chemoreceptor trimer, 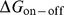, for the trimer orientation [19], [20] and separation [19], [22] observed in chemoreceptor lattices (Fig. 5B). Consistent with our results for a single trimer (S7 Figure) we find that there is a substantial membrane contribution to the transition energy of trimers in chemoreceptor lattices. Since chemoreceptors are functionally required to operate near zero transition energy [89], this membrane contribution must be compensated by internal protein contributions to the transition energy. However, Fig. 5B also shows that membrane-mediated interactions can lower the transition energy by up to approximately 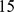 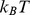 depending on the activity state of neighboring trimers, or by approximately 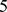 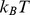 for each nearest-neighbor trimer in the off state. This cooperative shift in the transition energy is comparable to the shift in the trimer transition energy obtained by methylation of all 24 modification sites on a trimer [88], and may therefore be relevant for the cooperative signaling properties of chemoreceptor lattices. Furthermore, Fig. 5B shows that the strength of the predicted cooperative interactions among chemoreceptor trimers is robust with respect to variations in hydrophobic mismatch (Fig. 5B main panel) and membrane tension (Fig. 5B inset).

## Discussion

Fluorescence experiments have suggested a stochastic model for chemoreceptor lattice formation [28], [30] in which self-assembly of chemoreceptor lattices proceeds by nucleation and growth, without direct cytoskeletal involvement or active transport. Lattices consist of trimers-of-dimers of chemoreceptors and require the linker/kinase CheA and the linker CheW [19], [20] for their function. However, clustering of chemoreceptor trimers requires neither CheA nor CheW [27], [28]. In common with other membrane proteins [31], [32], chemoreceptor trimers are expected to deform the surrounding lipid bilayer, leading to membrane-mediated interactions [33], [34] between neighboring trimers. To quantify the role of membrane-mediated interactions in the assembly and architecture of chemoreceptor lattices we have developed a biophysical model of bilayer-chemoreceptor interactions.

Our biophysical model of bilayer-chemoreceptor interactions shows that membrane-mediated interactions yield attractive interactions between chemoreceptor trimers over several nanometers and hence provide a biophysical mechanism for cluster self-assembly. Our model predicts that the tip-on orientation of a pair of chemoreceptor trimers is a “gateway” state during assembly, whereas at smaller trimer separations, membrane-mediated interactions favor the face-on orientation of each trimer pair also observed in the presence of CheA and CheW [19], [20]. Furthermore, we predict that membrane-mediated interactions are strong enough to induce cluster formation even for a trimer concentration of only 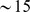 trimers per *E. coli* cell. This suggests a scenario for self-assembly in which membrane-mediated interactions produce clusters of chemoreceptors, which are further stabilized and ordered through protein interactions mediated by CheA and CheW. Since the range of membrane-mediated interactions is set by the elastic decay length of thickness deformations, which is a bilayer property, these conclusions do not rely on the detailed size and shape of trimers in our model of chemoreceptor trimers in Fig. 1B. In particular, for a given bilayer membrane the range of membrane-mediated interactions between trimers, measured in terms of the center-to-center distance between trimers, is determined by the edge-to-edge separation of trimers for each trimer configuration, yielding a longer (shorter) range of membrane-mediated interactions for larger (smaller) trimer sizes. In agreement with experimental observations [30], the strongly favorable interactions between trimers at small separations in Figs. 2 and 3 are expected to yield an approximately exponential size distribution of chemoreceptor clusters [30], [90].

*In vivo* electron cryo-tomography has revealed [19]–[25] that chemoreceptor lattices are not close-packed hexagonal arrays. Instead, chemoreceptor trimers form honeycomb lattices with a trimer at each vertex (S4B Figure), and a well-defined face-on orientation of trimers [19], [20]. Our model predicts that membrane-mediated interactions favor this face-on, honeycomb architecture of the lattice. In particular, we find that the three-fold symmetry and directionality of membrane-mediated interactions favor a honeycomb lattice (three neighbors per trimer) over a close-packed hexagonal lattice (six neighbors per trimer). Thus, while interactions with CheA and CheW are expected to determine the observed separation of trimers in chemoreceptor lattices [19], [22] and are likely to be adequate to define the observed lattice symmetry [73], [91], we find that membrane-mediated interactions can drive the formation of diffuse, less ordered chemoreceptor clusters [27], [28] and further stabilize the face-on honeycomb architecture of chemoreceptor lattices involving CheA and CheW. These results rely only on generic properties of chemoreceptor trimers and the cytoplasmic membrane, specifically the three-fold symmetry of trimers and a hydrophobic mismatch between trimers and the cytoplasmic membrane. This generality suggests that membrane-mediated interactions may facilitate the consistently observed honeycomb architecture of chemoreceptor lattices [22].

Membrane-mediated interactions extend over a longer range than direct protein-protein interactions, but may be weaker in magnitude. Thus, membrane-mediated interactions in chemoreceptor lattices complement direct protein-protein interactions, yielding robustness of the overall chemoreceptor lattice architecture against local disruption. Indeed, it has been observed [9], [26], [27] that chemoreceptor lattices can exhibit variable stoichiometries of chemoreceptors, CheA, and CheW. Our model predicts that membrane-mediated interactions can help to establish the proper orientation of neighboring trimers and the overall honeycomb lattice symmetry even at suboptimal protein stoichiometries, and thereby help to preserve lattice symmetry and stability. Conversely, it has been found [83]–[85] that overexpression of chemoreceptors can yield a two-dimensional hexagonal rather than a honeycomb lattice of trimers. In agreement with these observations, our model reveals that the honeycomb lattice structure is favored by the directionality of membrane-mediated interactions at moderate trimer densities while the hexagonal lattice structure is favored at high chemoreceptor densities.

Our model of chemoreceptor trimers in Fig. 1 assumes that chemoreceptor trimers induce bilayer deformations and possess a three-fold symmetry. The former assumption is thought [31], [32] to be a generic feature of transmembrane proteins such as chemoreceptors. The latter assumption is only justified if the three-fold symmetry of trimers, observed most directly in the cytoplasmic region of trimers, is also present in the transmembrane region of trimers. Electron cryo-tomography of chemoreceptor trimers has suggested [19], [20], [73] that the chemoreceptor dimers forming a trimer spread apart within the membrane. This may allow penetration of lipids into chemoreceptor trimers and, hence, membrane-mediated interactions within trimers. We did not consider such interactions here. Instead, we focused on membrane-mediated interactions between trimers which, within our model, might either correspond to compact chemoreceptor complexes or, alternatively, to lipid-chemoreceptor complexes. We note, however, that the penetration of lipids into chemoreceptor trimers may facilitate fluctuations in the relative positions of dimers within trimers, thereby reducing the rigidity of trimer shape. Such fluctuations could have interesting effects. For instance, while fluctuations in trimer shape are expected to reduce the directionality of membrane-mediated interactions, they could also increase the strength of membrane-mediated interactions by allowing a more favorable interface between neighboring trimers. Similarly, fluctuations in the structure of chemoreceptor trimers in the cyto- or periplasmic trimer regions could give rise to direct trimer-trimer interactions, which would compete with membrane-mediated interactions between trimers.

Our model of bilayer-chemoreceptor interactions suggests that localization of large chemoreceptor lattices to the cell poles is simply a consequence of the conical shape of individual chemoreceptor trimers [87], and neither requires interactions with CheA and CheW [27] nor curvature-mediated interactions among trimers. In agreement with experimental observations [21]–[25], [27]–[30], our model implies that large chemoreceptor clusters will tend to localize at the cell poles, while smaller chemoreceptor clusters can be distributed throughout the midcell regions. This mechanism for localization of large chemoreceptor lattices due to curvature sensing by individual chemoreceptor trimers is to be contrasted with a previously proposed mechanism [86] which assumes that trimers interact to yield a non-zero global intrinsic curvature of chemoreceptor lattices. A distinguishing difference between the localization mechanism proposed here and in Ref. [86] is that, according to the latter, chemoreceptor clusters should have a finite characteristic size set by the energy balance between short-range attraction and curvature-mediated long-range repulsion between trimers, whereas our model indicates that curvature-mediated interactions are too weak to limit cluster size in the absence of CheA and CheW. Fluorescence experiments [28], [30] measuring chemoreceptor cluster size in the absence of CheA and CheW may be able to distinguish between these two related scenarios for curvature-driven localization of large chemoreceptor lattices.

FRET experiments have revealed [8], [9] that chemoreceptors signal in cooperative teams of coupled trimers [9], [11]–[16]. Cooperative interactions among neighboring trimers are believed to be mediated by CheA and CheW [9], [10], [19], [20]. Our model of chemotactic signaling shows that, provided there is a substantial change in chemoreceptor hydrophobic thickness upon signaling, membrane-mediated interactions between chemoreceptor trimers [85] can in principle yield cooperative interaction energies of the order of several 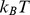. This would be sufficient [92] to account at least in part for the observed cooperative signaling properties of chemoreceptor lattices. Indeed, electron cryo-tomography indicates that honeycomb lattices of chemoreceptor trimers are somewhat disordered, with the degree of disorder being a matter of debate [21]–[25]. While interactions between chemoreceptor trimers mediated by CheA and CheW [9], [10], [19], [20] rely on a regular lattice structure, membrane-mediated interactions are less sensitive to defects in the chemoreceptor lattice. Thus, membrane-mediated interactions may increase the robustness of cooperative signaling teams, and complement cooperative interactions mediated by CheA and CheW [9], [10], [19], [20]. Consistent with our biophysical model of chemotactic signaling it has been found using homo-FRET [87], [93] that the *in vivo* signaling response of chemoreceptors depends on membrane-mechanical properties such as membrane tension. However, homo-FRET has so far not produced any evidence for cooperativity among chemoreceptor trimers in the absence of CheA and CheW [94]. Chemoreceptor clusters formed in the absence of CheA and CheW are more diffuse than chemoreceptor lattices formed in the presence of CheA and CheW [27], [28], which may substantially reduce membrane-mediated cooperativity.

Our model of chemotactic signaling predicts that shifts in the membrane contribution to the total free-energy difference between on and off states of chemoreceptor trimers due to changes in membrane composition or membrane tension can be comparable to shifts in the chemoreceptor transition energy due to receptor methylation [88], and can therefore be functionally relevant. In agreement with these predictions, it has been found that modifying the composition of lipid bilayers [35], [36] or bilayer-chemoreceptor interface [37] affects chemotactic signaling. In particular, changes in lipid composition can strongly bias chemoreceptors towards the active or inactive state [35], [36], and the baseline signaling state of chemoreceptors can be controlled by site-directed mutagenesis of chemoreceptor transmembrane helices [37]. Thus, in analogy to gramicidin [47], [71] and mechanosensitive [49], [72] channels, systematic variation of the membrane lipid composition, the chemoreceptor hydrophobic thickness, or membrane-mechanical properties such as membrane tension may allow quantitative experimental tests of our biophysical model of the role of membrane-mediated interactions in the assembly and architecture of chemoreceptor lattices, as well as our speculation of a membrane-mediated contribution to chemotactic signaling and cooperativity.

## Supporting Information

S1 Figure
**Schematic of midplane deformations induced by chemoreceptor trimers.** To complement the model of membrane-mediated interactions in Fig. 1 of the main text, we have estimated the midplane deformations induced by chemoreceptor trimers. Trimers can deform the bilayer midplane 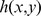 (dashed red line) by an angle 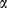 at the bilayer-trimer interface, and membrane-mediated interactions tilt trimers by an angle 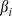 in the 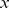-direction.(TIF)

S2 Figure
**Thickness deformation fields of chemoreceptor trimers.** Thickness deformations 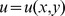 induced by two chemoreceptor trimers in (A) the tip-on and (B) the face-on orientation. Both chemoreceptor trimers are in the on state. (See also Fig. 1B of the main text.)(TIF)

S3 Figure
**Thickness deformation profile around a cylindrical membrane inclusion.** The thickness deformation field 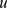 is calculated using the same parameter values as in Fig. 2 of the main text, but for a single cylindrical membrane inclusion of radius 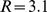 nm. The variable 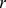 denotes the distance from the inclusion boundary and is measured in units of thickness deformation decay length 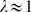 nm. The thickness deformation field 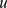 is measured in units of hydrophobic mismatch 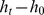.(TIF)

S4 Figure **Schematic of chemoreceptor lattice symmetries.** (A) Hexagonal lattice. (B) Face-on honeycomb lattice. (C) Tip-on honeycomb lattice.
(TIF)

S5 Figure
**Membrane-mediated interactions in chemoreceptor lattices.** Calculated elastic interaction energy per trimer, 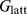, in face-on honeycomb (blue), tip-on honeycomb (red), and hexagonal (black) lattices as a function of (A) center-to-center distance between neighboring trimers (data as in Fig. 4A of the main text and shown here for completeness) and (B,C,D) monolayer hydrophobic thickness and membrane tension (insets) at 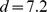 nm, 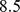 nm, and 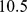 nm, as indicated by arrows in (A). The solid vertical line at 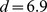 nm in (A) shows the trimer separation observed in face-on honeycomb chemoreceptor lattices [19], [22], and dashed vertical lines in (A) indicate steric constraints on lattice configurations. As in Fig. 4A of the main text, we assumed for (A) a membrane with 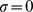 and a monolayer thickness 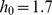 nm corresponding to the *E. coli* cytoplasmic membrane (shown by dashed vertical lines in (B–D)). For the main panels in (B–D) we set 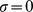 and the insets in (B–D) were obtained with 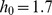 nm. All lattice energies were calculated from pairwise nearest-neighbor interaction potentials as shown in Fig. 2 of the main text.(TIF)

S6 Figure
**Effect of higher-order interactions on lattice energies.** Lattice energies for face-on honeycomb, tip-on honeycomb, and hexagonal lattices of chemoreceptor trimers allowing for up to nearest-neighbor (solid curves; as in Fig. 4A of the main text), next-nearest neighbor (dashed curves), and next-next-nearest neighbor (dotted-dashed curves) interactions. For each lattice symmetry, nearest neighbors, next-nearest neighbors, and next-next-nearest neighbors are indicated by the color coding in the inset, with 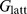 corresponding to the central trimer with white filling. All lattice energies were calculated from pairwise interaction potentials as shown in Fig. 2 of the main text.(TIF)

S7 Figure
**Membrane contribution to the transition energy of a single chemoreceptor trimer.** For the main panel we set 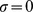 and for the inset we used the value 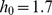 nm corresponding to the *E. coli* cytoplasmic membrane.(TIF)

S1 Video
**Video of gateway to assembly of face-on trimer configuration.** Gateway states in Fig. 3 of the main text for the assembly of the face-on trimer configuration at 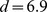 nm [19], [22].(AVI)

S1 Text **SI sections 1–3**.
(PDF)
