# Supplementary figures and images for: The Role of Membrane-Mediated Interactions in the Assembly and Architecture of Chemoreceptor Lattices
Source: PLoS Comput Biol. 2014 Dec 11;10(12):e1003932. doi: 10.1371/journal.pcbi.1003932 (PMC4263354; doi:10.1371/journal.pcbi.1003932)

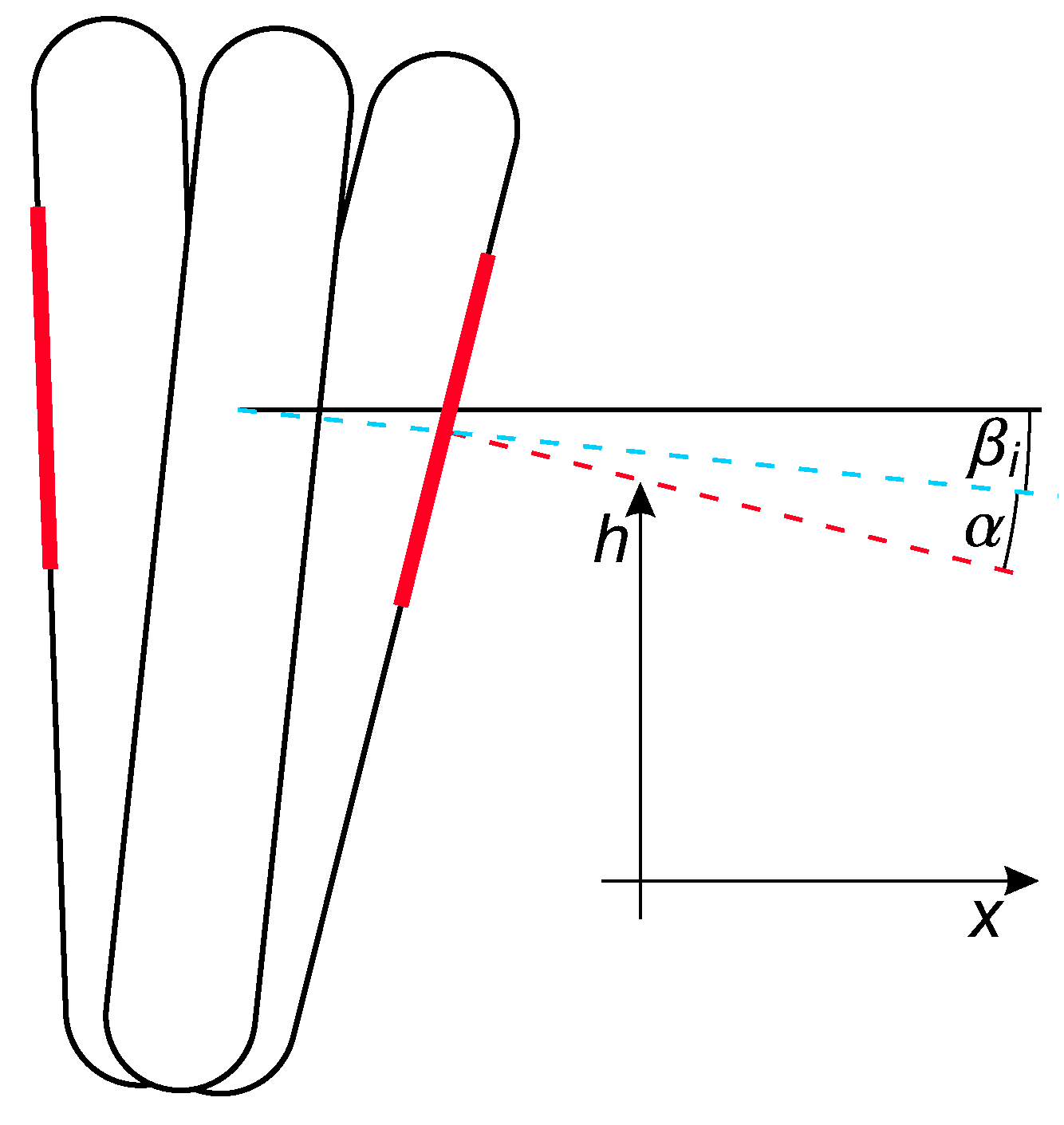

Supplement: S1 Figure — Schematic of midplane deformations induced by chemoreceptor trimers. To complement the model of membrane-mediated interactions in Fig. 1 of the main text, we have estimated the midplane deformations induced by chemoreceptor trimers. Trimers can deform the bilayer midplane (dashed red line) by an angle at the bilayer-trimer interface, and membrane-mediated interactions tilt trimers by an angle in the -direction. (TIF) [file pcbi.1003932.s001.tif]

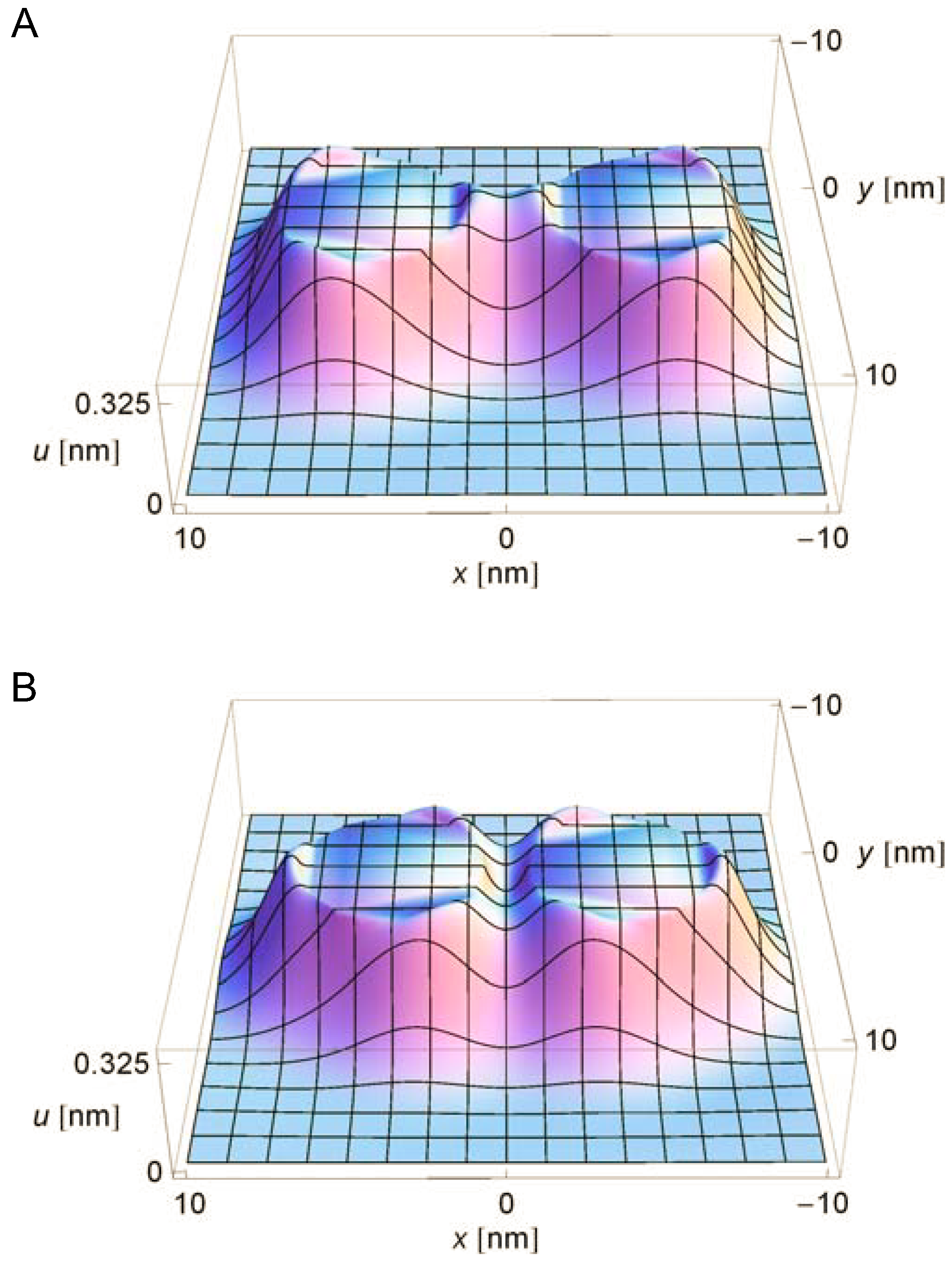

Supplement: S2 Figure — Thickness deformation fields of chemoreceptor trimers. Thickness deformations induced by two chemoreceptor trimers in (A) the tip-on and (B) the face-on orientation. Both chemoreceptor trimers are in the on state. (See also Fig. 1B of the main text.) (TIF) [file pcbi.1003932.s002.tif]

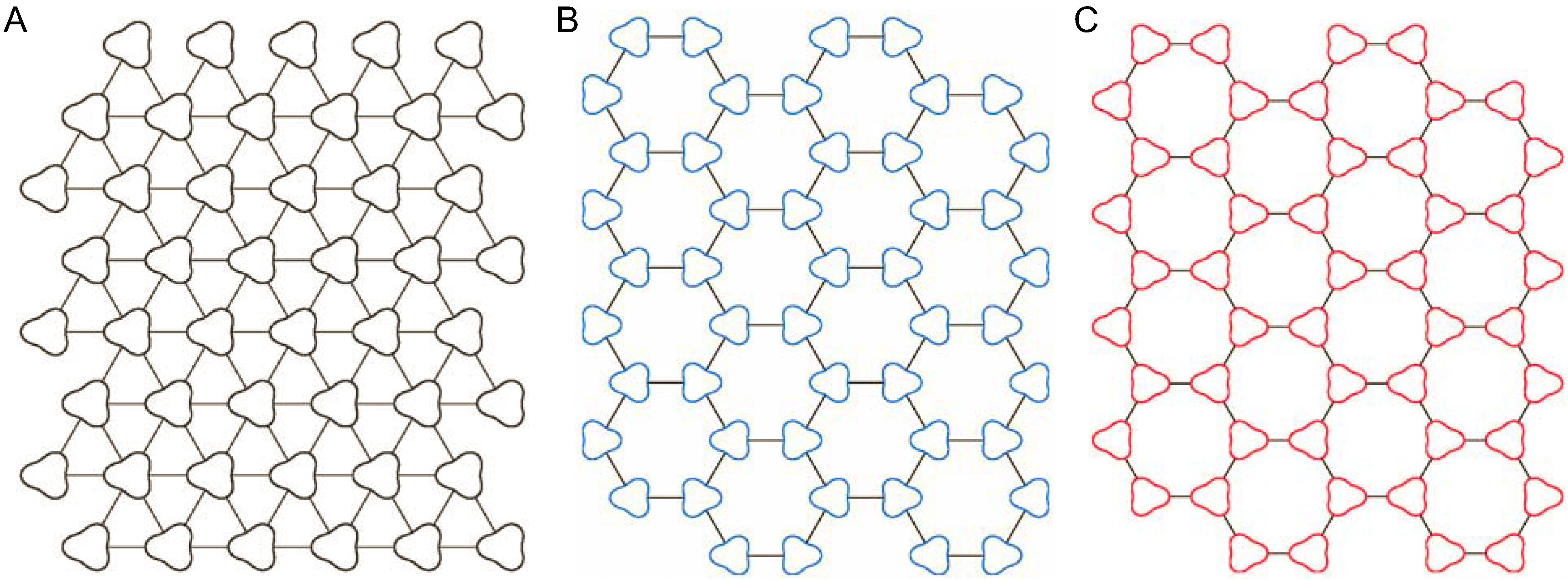

Supplement: S4 Figure — Schematic of chemoreceptor lattice symmetries. (A) Hexagonal lattice. (B) Face-on honeycomb lattice. (C) Tip-on honeycomb lattice. (TIF) [file pcbi.1003932.s004.tif]

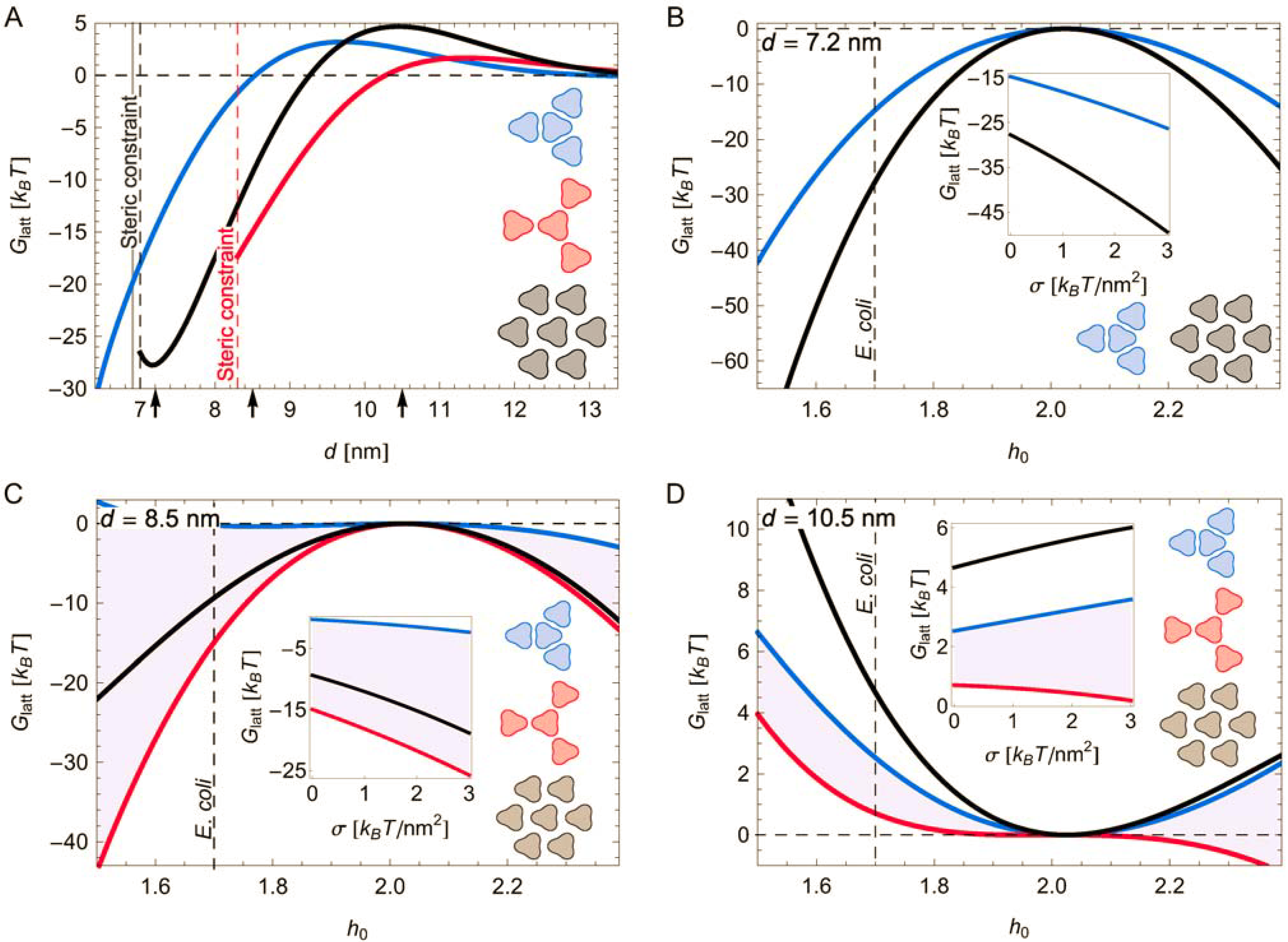

Supplement: S5 Figure — Membrane-mediated interactions in chemoreceptor lattices. Calculated elastic interaction energy per trimer, , in face-on honeycomb (blue), tip-on honeycomb (red), and hexagonal (black) lattices as a function of (A) center-to-center distance between neighboring trimers (data as in Fig. 4A of the main text and shown here for completeness) and (B,C,D) monolayer hydrophobic thickness and membrane tension (insets) at nm, nm, and nm, as indicated by arrows in (A). The solid vertical line at nm in (A) shows the trimer separation observed in face-on honeycomb chemoreceptor lattices [19], [22], and dashed vertical lines in (A) indicate steric constraints on lattice configurations. As in Fig. 4A of the main text, we assumed for (A) a membrane with and a monolayer thickness nm corresponding to the E. coli cytoplasmic membrane (shown by dashed vertical lines in (B–D)). For the main panels in (B–D) we set and the insets in (B–D) were obtained with nm. All lattice energies were calculated from pairwise nearest-neighbor interaction potentials as shown in Fig. 2 of the main text. (TIF) [file pcbi.1003932.s005.tif]

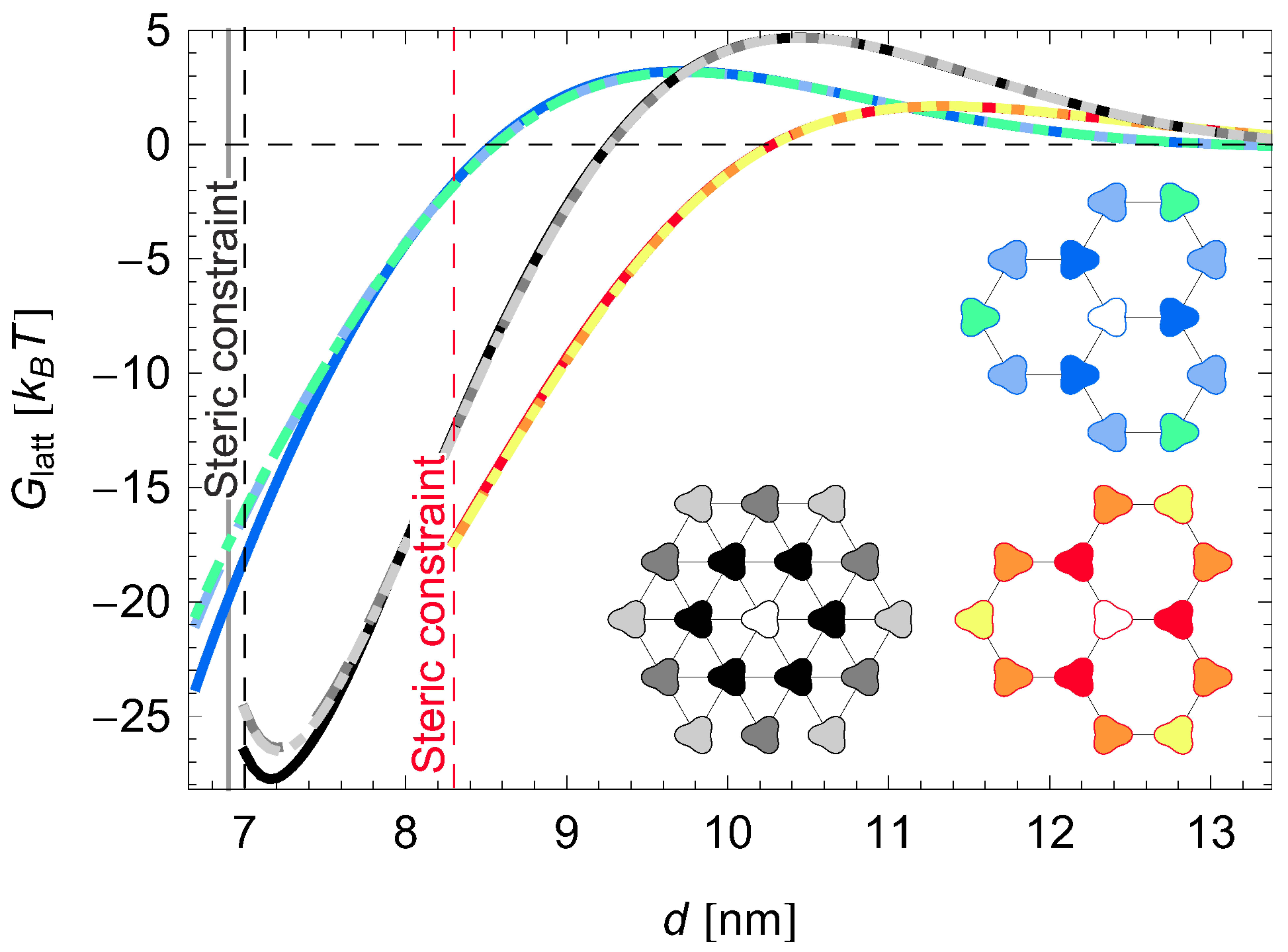

Supplement: S6 Figure — Effect of higher-order interactions on lattice energies. Lattice energies for face-on honeycomb, tip-on honeycomb, and hexagonal lattices of chemoreceptor trimers allowing for up to nearest-neighbor (solid curves; as in Fig. 4A of the main text), next-nearest neighbor (dashed curves), and next-next-nearest neighbor (dotted-dashed curves) interactions. For each lattice symmetry, nearest neighbors, next-nearest neighbors, and next-next-nearest neighbors are indicated by the color coding in the inset, with corresponding to the central trimer with white filling. All lattice energies were calculated from pairwise interaction potentials as shown in Fig. 2 of the main text. (TIF) [file pcbi.1003932.s006.tif]
