# Supplementary material for: The Role of Membrane-Mediated Interactions in the Assembly and Architecture of Chemoreceptor Lattices
Source: PLoS Comput Biol. 2014 Dec 11;10(12):e1003932. doi: 10.1371/journal.pcbi.1003932 (PMC4263354; doi:10.1371/journal.pcbi.1003932)
Supplement: S1 Text — SI sections 1–3. (PDF) [file pcbi.1003932.s009.pdf]

The Role of Membrane-Mediated Interactions in the Assembly and Architecture of Chemoreceptor Lattices

Supporting Information Text

Christoph A. Haselwandter[1] and Ned S. Wingreen[2]

[1]*Departments of Physics & Astronomy and Biological Sciences, University of Southern California, Los Angeles, CA 90089, USA*

[2]*Department of Molecular Biology, Princeton University, Princeton, NJ 08544, USA*

1 Analytic calculation of membrane-mediated interactions

As described in the main text, our biophysical model of membrane-mediated interactions between chemoreceptor trimers is based on the standard framework of membrane mechanics [1–8], in which the heights of the hydrophilic-hydrophobic interfaces at coordinates (x, y) in the top and bottom (outer and inner) membrane leaflets are represented by the functions $h_+(x, y)$ and $h_-(x, y)$. It is convenient to express the fields $h_+(x, y)$ and $h_-(x, y)$ in terms of the thickness deformation field $u(x, y)$ (Eq. (1) of the main text) and the midplane deformation field $h(x, y)$:

$$h(x, y) = \frac{1}{2}\left[h_+(x, y) + h_-(x, y)\right] . \tag{S1}$$

To leading order in u and h, the total elastic energy of the bilayer-trimer system, $G[u, h]$, can then be written as the sum of two decoupled free energies associated with midplane and thickness deformations:

$$G[u, h] = G_u[u] + G_h[h] , \tag{S2}$$

in which $G_u[u]$ is given in Eq. (2) of the main text and

$$G_h[h] = \frac{1}{2}\int dxdy \left[K_b \left(\nabla^2 h\right)^2 + \sigma(\nabla h)^2\right] , \tag{S3}$$

where, as discussed in the main text, the parameters K_b and σ are the bending rigidity and the membrane tension, respectively.

Energetic contributions to membrane-mediated protein interactions due to thickness deformations, G_u, are expected [7] to dominate over energetic contributions due to midplane deformations, G_h, at the small trimer separations relevant for chemoreceptor lattices. We therefore focus here on thickness deformations. A discussion of midplane deformations is provided in Sec. 2. Following the standard membrane-mechanical framework for describing bilayer-protein interactions [3, 4, 7–31], the specific properties of chemoreceptor trimers enter our model of membrane-mediated interactions between chemoreceptor trimers through the boundary conditions at the bilayer-trimer interface. For thickness deformations, the boundary conditions associated with each chemoreceptor trimer i, where $i = 1, 2, \ldots$, take the general form

$$u(r_i, \theta_i)\big|_{r_i=C_i} = u_t , \tag{S4}$$

$$\hat{\mathbf{n}} \cdot \nabla u(r_i, \theta_i)\big|_{r_i=C_i} = u'_t , \tag{S5}$$

where $\hat{\mathbf{n}}$ is the unit normal vector along the bilayer-trimer boundary, r_i and θ_i are the radial coordinate and polar angle associated with a polar coordinate system having trimer i at its center, and $C_i = C_i(\theta_i)$ describes the shape of trimer i as a function of θ_i. A simple coarse-grained model of the hydrophobic shape of a chemoreceptor trimer (which may correspond either to compact chemoreceptors or, alternatively, to a lipid-chemoreceptor complex) is given by

$$C_i(\theta_i) = R\left[1 + \epsilon \cos 3\left(\theta_i - \omega_i\right)\right] , \tag{S6}$$

where R is the average trimer radius, ϵ is the perturbation amplitude, and the phase shift ω_i captures the orientation of trimer i. Based on images of chemoreceptor trimers obtained by electron cryo-tomography [32, 33] we estimated the parameter values $R = 3.1$ nm and $\epsilon = 0.2$. Equation (S6) is illustrated in Figure 1B of the main text.

The trimer hydrophobic mismatch u_t in Eq. (S4) plays a crucial role in our model, and is related to the trimer hydrophobic thickness h_t by

$$u_t = h_t - h_0 \,. \tag{S7}$$

A typical value of h_0 for the *E. coli* cytoplasmic membrane is $h_0 = 1.7$ nm [21, 34] while, for example, the approximate hydrophobic thickness of the chemoreceptor Trg is $h_t = 2.025$ nm [35], which we used for all the calculations described here. In general, the value of h_0 can be tuned by changing the membrane composition. In the main text we therefore study membrane-mediated interactions between chemoreceptor trimers as a function of u_t and, hence, of h_t and h_0. In the membrane-mechanical model of bilayer-protein interactions, the value of u_t' in Eq. (S5) is found [9, 11, 19, 20] to play a minor role compared to the value of u_t in Eq. (S4), and u_t' is often set to zero. Following these previous studies, we also set $u_t' = 0$ in our calculations.

Interaction potentials between chemoreceptor trimers are obtained by minimizing $G_u[u]$ in Eq. (2) of the main text subject to the constraints in Eqs. (S4) and (S5). This is achieved by following the analytic methodology developed in Ref. [36]. In particular, the general analytic series solution for the energetic cost of membrane-mediated interactions between proteins in Ref. [36] is easily adapted to describe membrane-mediated interactions between chemoreceptor trimers. For completeness, we summarize here the key steps. We first note that the Euler-Lagrange equation associated with Eq. (2) of the main text is given by

$$\left(\nabla^2 - \nu_+\right)\left(\nabla^2 - \nu_-\right)\bar{u} = 0 \,, \tag{S8}$$

where, for convenience, we have introduced the function

$$\bar{u}(x, y) = u(x, y) + \frac{\sigma h_0}{K_t} \,, \tag{S9}$$

and

$$\nu_\pm = \frac{1}{2K_b}\left[\sigma \pm \left(\sigma^2 - \frac{4K_bK_t}{h_0^2}\right)^{1/2}\right] . \tag{S10}$$

We analytically solve Eq. (S8) for interacting chemoreceptor trimers by making the ansatz [24, 26]

$$\bar{u} = \bar{u}_1(r_1, \theta_1) + \bar{u}_2(r_2, \theta_2) \,, \tag{S11}$$

where the $\bar{u}_i(r_i, \theta_i)$ are the general solutions of Eq. (S8) appropriate for a single trimer [37] centered at $r_i = 0$. We find

$$\bar{u}_i(r_i, \theta_i) = f_i^+(r_i, \theta_i) + f_i^-(r_i, \theta_i) \,, \tag{S12}$$

which can be written as a Fourier-Bessel series [36]

$$f_i^\pm(r_i, \theta_i) = A_{i,0}^\pm K_0(\sqrt{\nu_\pm} r_i) + \sum_{n=1}^{\infty}\left\{A_{i,n}^\pm K_n(\sqrt{\nu_\pm} r_i)\cos n\theta + B_{i,n}^\pm K_n(\sqrt{\nu_\pm} r_i)\sin n\theta\right\} , \tag{S13}$$

in which the K_n are modified Bessel functions of the second kind, and we have assumed [11] that thickness deformations decay away from single trimers. The constants $A_{i,n}^\pm$ and $B_{i,n}^\pm$ in Eq. (S13) are fixed [36] by expanding $\bar{u}$ in the vicinity of each trimer in the small parameter R/d, where d is the center-to-center distance between trimers, expanding Eqs. (S4) and (S5) in the small parameter ϵ in Eq. (S6) [27, 31], and matching the solution $\bar{u}$ at each order in the Fourier-Bessel series to the boundary conditions. Finally, we analytically calculate the interaction energy between two trimers by using Eq. (S8) to transform [36, 37] the surface integral in Eq. (2) of the main text to a sum of line integrals along the bilayer-trimer boundaries, which are then evaluated at each order in the Fourier-Bessel series in Eq. (S11).

2 Midplane deformations

The midplane deformations induced by chemoreceptor trimers can be captured by the midplane bending energy in Eq. (S3). The Euler-Lagrange equation associated with Eq. (S3) is given by

$$K_b \nabla^4 h - \sigma \nabla^2 h = 0\,. \tag{S14}$$

Similar to the case of thickness deformations, the specific properties of chemoreceptor trimers enter Eq. (S14) through the boundary conditions at the bilayer-trimer interface. Following Refs. [24, 26] we estimate the midplane deformations induced by chemoreceptor trimers by employing the boundary conditions

$$h|_{r_i=R} = H_i + R\beta_i \cos\theta_i\,, \tag{S15}$$

$$\left.\frac{\partial h}{\partial r_i}\right|_{r_i=R} = \alpha + \beta_i \cos\theta_i\,, \tag{S16}$$

where we use the same notation as in Sec. 1 and, for simplicity, we have approximated chemoreceptor trimers as conical inclusions of radius R at the bilayer midplane.

The parameter α in Eq. (S16) is the contact angle between trimers and the midplane of the surrounding lipid bilayer (Figure S1). A non-zero value of α corresponds to a conical shape of chemoreceptor trimers with apex angle 2α. For $\alpha \neq 0$ the up-down symmetry of the membrane is broken, resulting in midplane deformations of the bilayer membrane. On the basis of the available structural information on chemoreceptor trimers Vaknin and Berg [38] estimated $\alpha \approx 0.17$ rad. The parameters H_i and β_i in Eqs. (S15) and (S16) allow for the center of each trimer to be located at some height H_i above the x-y plane, and for the boundary of each trimer to be tilted by some angle β_i in the x-direction (Figure S1). The values of H_i and β_i are not arbitrary but, rather, are determined by conditions of zero vertical force and zero torque on each trimer [26]:

$$\int_0^{2\pi} d\theta_i \left[R \frac{\partial}{\partial r_i} \left(\sigma h - K_b \nabla^2 h \right) \right]_{r_i=R} = 0\,, \tag{S17}$$

$$\int_0^{2\pi} d\theta_i \cos\theta_i \left[R^2 \frac{\partial}{\partial r_i} \left(\sigma h - K_b \nabla^2 h \right) + K_b R \nabla^2 h \right]_{r_i=R} = 0\,. \tag{S18}$$

Following Ref. [26] we assume that membrane deformations decay to zero away from chemoreceptor trimers, which means that $\nabla h \to 0$ for $r_i \to \infty$. Equations (S15)–(S18) then fully specify the solution of Eq. (S14) and, hence, the midplane deformation energy of interacting chemoreceptor trimers in Eq. (S3).

Midplane interactions are weak

The membrane-mediated interactions between chemoreceptor trimers due to midplane deformations in Eq. (S3) compete with the membrane-mediated interactions due to thickness deformations in Eq. (2) of the main text. How important are midplane deformations from an energetic perspective? Similar to the case of thickness deformations, we employed the analytic methodology summarized in Sec. 1 and described further in Ref. [36] to calculate interaction potentials between chemoreceptor trimers due to midplane deformations. Consistent with previous studies [7, 24, 26] we find that, in contrast to membrane-mediated interactions due to thickness deformations, midplane deformations induce repulsion between trimers in chemoreceptor lattices. For the trimer separation and orientation observed in experiments [32, 33, 39], Eqs. (S3) and (S14) yield the interaction strength

$$G_h^{\text{int}} \approx 0.4 K_b \alpha^2 \tag{S19}$$

at $\sigma = 0$ for the value $R = 2.48$ nm corresponding to the face-on orientation of chemoreceptor trimers observed in experiments [32, 33]. Using the typical membrane bending rigidity $K_b = 20\ k_BT$ [21] and the value $\alpha = 0.17$ rad estimated for chemoreceptor trimers [38], Eq. (S19) gives $G_h^{\text{int}} \approx 0.3\ k_BT$. Thus, the midplane interaction strength is well below k_BT and typically more than an order of magnitude smaller than the interaction strength associated with thickness deformations. In the main text we therefore focus on thickness deformations as the dominant physical mechanism for membrane-mediated interactions between chemoreceptor trimers.

Localization of large chemoreceptor lattices to the cell poles

The overall energetic cost of trimer-induced midplane deformations depends on the interplay between the conical shape of chemoreceptor trimers and the preferred curvature of the surrounding lipid bilayer membrane. In particular, the membrane radius of curvature at the poles of *E. coli* is twice that of the midcell region, and has the same sign as the radius of curvature of chemoreceptor trimers (Figure S1). Can the midplane deformations induced by chemoreceptor trimers act as curvature sensors driving the localization of trimers to the cell poles? Indeed, assuming that chemoreceptor lattices possess an intrinsic curvature it was found [40] that it is energetically favorable for chemoreceptor lattices to be localized at the cell poles. We argue below that, even if chemoreceptor trimers do not interact to produce a non-zero intrinsic curvature of chemoreceptor lattices, bilayer-trimer interactions can yield localization of large chemoreceptor lattices to the cell poles.

A simple estimate of the energetic cost of midplane deformations induced by individual chemoreceptor trimers is given by [20, 21]

$$G_h \sim \pi R \sqrt{\sigma K_b} \alpha_{\text{eff}}^2 \,, \tag{S20}$$

where α_{eff} is the effective membrane contact angle between a chemoreceptor trimer and the surrounding lipid bilayer. Equation (S20) is derived [21] by reducing the problem of calculating the two-dimensional midplane deformation field in Eqs. (S3) and (S14) to a one-dimensional variational problem. A rough estimate of the effect of the preferred membrane curvature on midplane deformations is obtained by setting

$$\alpha_{\text{eff}} = \alpha - \gamma \,, \tag{S21}$$

in which

$$\gamma = \arcsin \frac{R}{c^*} \,, \tag{S22}$$

where c^* is the preferred membrane radius of curvature set by the cell wall, with $c^*_{\text{pole}} \approx 0.4\ \mu$m for the poles and $c^*_{\text{midcell}} \approx 0.8\ \mu$m for the midcell of *E. coli* [1, 2]. Equation (S21) corresponds to tilting the one-dimensional coordinate system in Figure S1 by an angle γ, and replacing α by α_{eff}.

The midplane deformation energy in Eq. (S20) considers the effects of membrane tension and membrane bending, but does not allow for interactions between deformations in the cytoplasmic membrane and the cell wall. In living *E. coli* such interactions constrain midplane deformations by pinning the cytoplasmic membrane to the cell wall [41, 42]. The effects of membrane pinning on trimer-induced midplane deformations can be modeled by adding to the midplane deformation energy in Eq. (S3) the term [41, 42]

$$G_h^{\text{pin}} = \frac{K_p}{2} \int dxdy \, (h - h_p)^2 \,, \tag{S23}$$

where K_p is the pinning modulus and $h_p(x, y)$ describes the preferred shape of the cell membrane set by the cell wall. If ∇h_p and $\nabla^2 h_p$ are small compared to ∇h and $\nabla^2 h$, one can fix [40] the membrane height so that $h_p(x, y) = 0$ in Eq. (S23) while leaving Eq. (S3) unchanged. If membrane pinning dominates over membrane tension, $K_p \gg \sigma^2/K_b$, Eqs. (S3) and (S23) yield the midplane deformation

energy

$$G_h \sim \pi R \left(\frac{5 K_b K_p^{1/3}}{3} \right)^{3/4} \alpha_{\text{eff}}^2 \,, \tag{S24}$$

where α_{eff} is defined in Eq. (S21). Equation (S24) provides an estimate of the midplane deformation energy of chemoreceptor trimers in the presence of strong membrane pinning. We derived Eq. (S24) by a one-dimensional variational approach following similar steps [21] as for Eq. (S20).

For $K_b = 20\ k_B T$ [21], the average value $R = 3.1$ nm, and $\alpha = 0.17$ rad [38], Eqs. (S20) and (S24) both yield an energy difference $\Delta G_{\text{midcell}-\text{pole}} \lesssim 0.13\ k_B T$ between the midcell and poles of *E. coli*, where we have used the typical parameter values $\sigma = 1\ k_B T/\text{nm}^2$ [7] in Eq. (S20) and $K_p = 0.28\ k_B T/\text{nm}^4$ [41, 42] in Eq. (S24). These estimates suggest that midplane deformations are not able to localize single chemoreceptor trimers to the poles of *E. coli.* However, as shown in the main text, membrane-mediated interactions due to thickness deformations can yield strong attraction between chemoreceptor trimers, resulting in large chemoreceptor lattices. In the limit of weak interactions due to midplane deformations, Eqs. (S20) and (S24) yield

$$0.055 N k_B T \lesssim \Delta G_{\text{midcell}-\text{pole}} \lesssim 0.13 N k_B T \tag{S25}$$

for a lattice composed of N chemoreceptor trimers, where the lower bound corresponds to Eq. (S20) and the upper bound corresponds to Eq. (S24). Thus, it is energetically favorable for chemoreceptor lattices to be localized in curved, convex membrane regions such as provided by the cell poles. Note that this physical mechanism for the localization of chemoreceptor lattices only becomes effective at large enough lattice sizes. Large chemoreceptor lattices composed of thousands of receptors (yielding $\Delta G_{\text{midcell}-\text{pole}} \gtrsim 20\ k_B T$ in Eq. (S25)) are indeed observed predominantly at the cell poles, while smaller chemoreceptor lattices are also found in the midcell regions [39, 43–50]. The estimates in Eq. (S25) indicate that bilayer-trimer interactions are sufficient to localize large chemoreceptor lattices to convex regions of the cell membrane and that, in particular, localization of large chemoreceptor lattices to the cell poles does not require a non-zero intrinsic curvature of chemoreceptor lattices. Indeed, membrane-mediated interactions between chemoreceptor trimers due to curvature deformations are expected to be weak and, with the cytoplasmic membrane pinned against the cell wall, approximately pairwise additive [42]. This suggests that curvature-mediated interactions are not able to yield a well-defined intrinsic curvature of chemoreceptor clusters in the absence of CheA and CheW, but rather that additive curvature sensing by individual trimers may drive localization of large chemoreceptor lattices to the cell poles.

3 Critical trimer concentration for chemoreceptor lattices

In the main text we found that trimer-induced thickness deformations of the bilayer membrane can yield strong attraction between chemoreceptor trimers. Membrane-mediated interactions due to thickness deformations therefore favor the formation of large chemoreceptor lattices. However, the localization of trimers in chemoreceptor lattices means that individual trimers are not free to diffuse in the cytoplasmic membrane, which incurs a free energy cost due to loss of entropy. Based on previous work [41, 42] on lipid clusters, we expect entropic effects to dominate if the trimer concentration in the membrane is low, yielding disperse trimers. For high enough trimer concentrations, however, attraction due to membrane-mediated interactions is expected to dominate over entropic effects, yielding chemoreceptor clusters.

The above considerations can be quantified by considering the critical fraction of membrane area occupied by trimers, ϕ_c, for which half of all trimers in the cytoplasmic membrane are dispersed and half are bound in lattices. In the regime of dilute trimer concentrations, a simple estimate of ϕ_c is provided by [42]

$$\phi_c \sim 2 e^{-|G_{\text{latt}}|/k_B T} \,, \tag{S26}$$

where, as in the main text, G_{latt} is the elastic interaction energy per trimer in chemoreceptor lattices. As discussed in the main text, the value of G_{latt} depends on membrane properties such as membrane tension and the hydrophobic mismatch between chemoreceptor trimers and the surrounding lipid bilayer. However, the results in the main text suggest $|G_{\mathrm{latt}}| \sim 10\ k_B T$ as an order of magnitude estimate of $|G_{\mathrm{latt}}|$ in *E. coli.* For $|G_{\mathrm{latt}}| = 10\ k_B T$, Eq. (S26) yields $\phi_c \sim 0.9 \times 10^{-4}$. Using the sphero-cylindrical model of the shape of *E. coli* [1,2] with an overall cell length of 2 μm and radius of curvature of 0.4 μm at the cell poles, and the approximate trimer area 30 nm^2 corresponding to the average trimer radius $R = 3.1$ nm, $\phi_c \sim 0.9 \times 10^{-4}$ implies that the critical trimer concentration is already reached with approximately 15 chemoreceptor trimers in the cytoplasmic membrane. Thus, based on Eq. (S26) we conclude that, even if trimers are very dilute in the cytoplasmic membrane, membrane-mediated interactions can yield formation of chemoreceptor clusters.

References

[1] Boal D (2002) Mechanics of the Cell. Cambridge: Cambridge University Press.

[2] Phillips R, Kondev J, Theriot J, Garcia HG (2013) Physical Biology of the Cell. London and New York: Garland Science, 2nd edition.

[3] Andersen OS, Koeppe RE II (2007) Bilayer thickness and membrane protein function: An energetic perspective. Annu Rev Biophys Biomol Struct 36: 107–120.

[4] Jensen MO, Mouritsen OG (2004) Lipids do influence protein function—the hydrophobic matching hypothesis revisited. Biochim Biophys Acta 1666: 205–226.

[5] Seifert U (1997) Configurations of fluid membranes and vesicles. Adv Phys 46: 13–137.

[6] Safran S (2003) Statistical Thermodynamics of Surfaces, Interfaces, and Membranes. Boulder: Westview Press.

[7] Phillips R, Ursell T, Wiggins P, Sens P (2009) Emerging roles for lipids in shaping membrane-protein function. Nature 459: 379–385.

[8] Fournier JB (1999) Microscopic membrane elasticity and interactions among membrane inclusions: interplay between the shape, dilation, tilt, and tilt-difference modes. Eur Phys J B 11: 261–272.

[9] Huang HW (1986) Deformation free energy of bilayer membrane and its effect on gramicidin channel lifetime. Biophys J 50: 1061–1070.

[10] Dan N, Pincus P, Safran SA (1993) Membrane-induced interactions between inclusions. Langmuir 9: 2768–2771.

[11] Nielsen C, Goulian M, Andersen OS (1998) Energetics of inclusion-induced bilayer deformations. Biophys J 74: 1966–1983.

[12] Dan N, Safran SA (1998) Effect of lipid characteristics on the structure of transmembrane proteins. Biophys J 75: 1410–1414.

[13] Aranda-Espinoza H, Berman A, Dan N, Pincus P, Safran S (1996) Interaction between inclusions embedded in membranes. Biophys J 71: 648–656.

[14] Brannigan G, Brown FLH (2006) A consistent model for thermal fluctuations and protein-induced deformations in lipid bilayers. Biophys J 90: 1501–1520.

[15] Brannigan G, Brown FLH (2007) Contributions of Gaussian curvature and nonconstant lipid volume to protein deformation of lipid bilayers. Biophys J 92: 864–876.

[16] Partenskii MB, Jordan PC (2002) Membrane deformation and the elastic energy of insertion: Perturbation of membrane elastic constants due to peptide insertion. J Chem Phys 117: 10768–10776.

[17] Partenskii MB, Miloshevsky GV, Jordan PC (2004) Membrane inclusions as coupled harmonic oscillators: Effects due to anistropic membrane slope relaxation. J Chem Phys 120: 7183–7193.

[18] Lundbæk JA (2006) Regulation of membrane protein function by lipid bilayer elasticity—a single molecule technology to measure the bilayer properties experienced by an embedded protein. J Phys: Condens Matter 18: S1305–S1344.

[19] Wiggins P, Phillips R (2004) Analytic models for mechanotransduction: Gating a mechanosensitive channel. Proc Natl Acad Sci USA 101: 4071–4076.

[20] Wiggins P, Phillips R (2005) Membrane-protein interactions in mechanosensitive channels. Biophys J 88: 880–902.

[21] Ursell T, Kondev J, Reeves D, Wiggins PA, Phillips R (2008) The role of lipid bilayer mechanics in mechanosensation. In: Kamkin A, Kiseleva I, editors, Mechanosensitivity in Cells and Tissues 1: Mechanosensitive Ion Channels. New York: Springer Press, pp. 37–70.

[22] Ursell T, Huang KC, Peterson E, Phillips R (2007) Cooperative gating and spatial organization of membrane proteins through elastic interactions. PLoS Comput Biol 3: e81.

[23] Harroun TA, Heller WT, Weiss TM, Yang L, Huang HW (1999) Theoretical analysis of hydrophobic matching and membrane-mediated interactions in lipid bilayers containing gramicidin. Biophys J 76: 3176–3185.

[24] Goulian M, Bruinsma R, Pincus P (1993) Long-range forces in heterogeneous fluid membranes. EPL 22: 145–150.

[25] Goulian M, Bruinsma R, Pincus P (1993) Long-range forces in heterogeneous fluid membranes. EPL 23: 155.

[26] Weikl TR, Kozlov MM, Helfrich W (1998) Interaction of conical membrane inclusions: Effect of lateral tension. Phys Rev E 57: 6988-6995.

[27] Kim KS, Neu J, Oster G (2000) Effect of protein shape on multibody interactions between membrane inclusions. Phys Rev E 61: 4281–4285.

[28] Kim KS, Neu J, Oster G (1998) Curvature-mediated interactions between membrane proteins. Biophys J 75: 2274-2291.

[29] Kim KS, Neu JC, Oster GF (1997) Many-body forces between membrane inclusions: A new pattern-formation mechanism. EPL 48: 99-105.

[30] Chou T, Kim KS, Oster G (2001) Statistical thermodynamics of membrane bending-mediated protein–protein attractions. Biophys J 80: 1075-1087.

[31] Kim KS, Chou T, Rudnick J (2008) Degenerate ground-state lattices of membrane inclusions. Phys Rev E 78: 011401.

[32] Briegel A, Li X, Bilwes A, Hughes K, Jensen GJ, et al. (2012) Bacterial chemoreceptor arrays are hexagonally-packed trimers of receptor dimers networked by rings of kinase and coupling proteins. Proc Natl Acad Sci USA 109: 3766-3771.

[33] Liu J, Hu B, Morado DR, Jani S, Manson MD, et al. (2012) Molecular architecture of chemoreceptor arrays revealed by cryoelectron tomography of *Escherichia coli* minicells. Proc Natl Acad Sci USA 109: E1481–E1488.

[34] Mitra K, Ubarretxena-Belandia I, Taguchi T, Warren G, Engelman DM (2004) Modulation of the bilayer thickness of exocytic pathway membranes by membrane proteins rather than cholesterol. Proc Natl Acad Sci USA 101: 4083-4088.

[35] Boldog T, Hazelbauer GL (2004) Accessibility of introduced cysteines in chemorceptor transmembrane helices reveals boundaries interior to bracketing charged residues. Prot Sci 13: 1466-1475.

[36] Haselwandter CA, Phillips R (2013) Directional interactions and cooperativity between mechanosensitive membrane proteins. EPL 101: 68002.

[37] Haselwandter CA, Phillips R (2013) Connection between oligomeric state and gating characteristics of mechanosensitive ion channels. PLoS Comput Biol 9: e1003055.

[38] Vaknin A, Berg HC (2006) Osmotic stress mechanically perturbs chemoreceptors in *Escherichia coli*. Proc Natl Acad Sci USA 103: 592-596.

[39] Briegel A, Ortega DR, Tocheva EI, Wuichet K, Li Z, et al. (2009) Universal architecture of chemoreceptor arrays. Proc Natl Acad Sci USA 106: 17181-17186.

[40] Endres RG (2009) Polar chemoreceptor clustering by coupled trimers of dimers. Biophys J 96: 453-463.

[41] Huang KC, Mukhopadhyay R, Wingreen NS (2006) A curvature-mediated mechanism for localization of lipids to bacterial poles. PLoS Comput Biol 2: e151.

[42] Mukhopadhyay R, Huang KC, Wingreen NS (2008) Lipid localization in bacterial cells through curvature-mediated microphase separation. Biophys J 95: 1034-1049.

[43] Shiomi D, Banno S, Homma M, Kawagishi I (2005) Stabilization of polar localization of a chemoreceptor via its covalent modifications and its communication with a different chemoreceptor. J Bacteriol 187: 7647-7654.

[44] Kentner D, Thiem S, Hildenbeutel M, Sourjik V (2006) Determinants of chemoreceptor cluster formation in *Escherichia coli*. Mol Microbiol 61: 407-417.

[45] Thiem S, Sourjik V (2008) Stochastic assembly of chemoreceptor clusters in *Escherichia coli*. Mol Microbiol 68: 1228-1236.

[46] Greenfield D, McEvoy AL, Shroff H, Crooks GE, Wingreen NS, et al. (2009) Self-organization of the *Escherichia coli* chemotaxis network imaged with super-resolution light microscopy. PLoS Biol 7: e1000137.

[47] Briegel AB, Ding HJ, Li Z, Gitai Z, Dias PD, et al. (2008) Location and architecture of the *Caulobacter crescentus* chemoreceptor array by electron cryotomography. Mol Microbiol 69: 30-41.

[48] Khursigara CM, Wu X, Subramanian S (2008) Chemoreceptors in *Caulobacter crescentus*: Trimers of receptor dimers in a partially ordered hexagonally packed array. J Bacteriol 190: 6805-6810.

[49] Khursigara CM, Lan G, Neumann S, Wu X, Ravindran S, et al. (2011) Lateral density of receptor arrays in the membrane plane influences sensitivity of the *E. coli* chemotaxis response. EMBO J 30: 1717-1729.

[50] Briegel A, Beeby M, Thanbichler M, Jensen GJ (2011) Activated chemoreceptor arrays remain intact and hexagonally packed. Mol Microbiol 82: 748-757.
